# Supplementary material for: Single-handed supramolecular double helix of homochiral bis(N-amidothiourea) supported by double crossed C−I···S halogen bonds
Source: Nat Commun. 2019 Aug 9;10:3610. doi: 10.1038/s41467-019-11539-5 (PMC6689071; doi:10.1038/s41467-019-11539-5)
Supplement: Supplementary file 1 — Supplementary Information [file 41467_2019_11539_MOESM1_ESM.pdf]

Supplementary Information

Single-handed supramolecular double helix of homochiral bis(*N*-amidothiourea) supported by double crossed C−I···S halogen bonds

Yan *et al.*

Supplementary Figures

Synthetic procedures

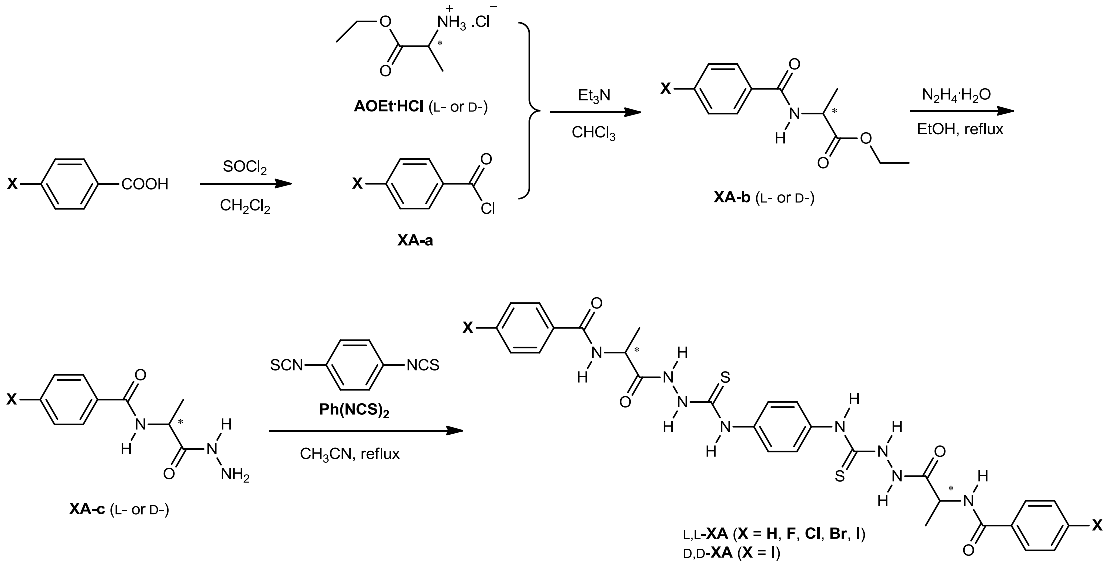

Supplementary Figure 1. General procedures for the syntheses of **XAs**

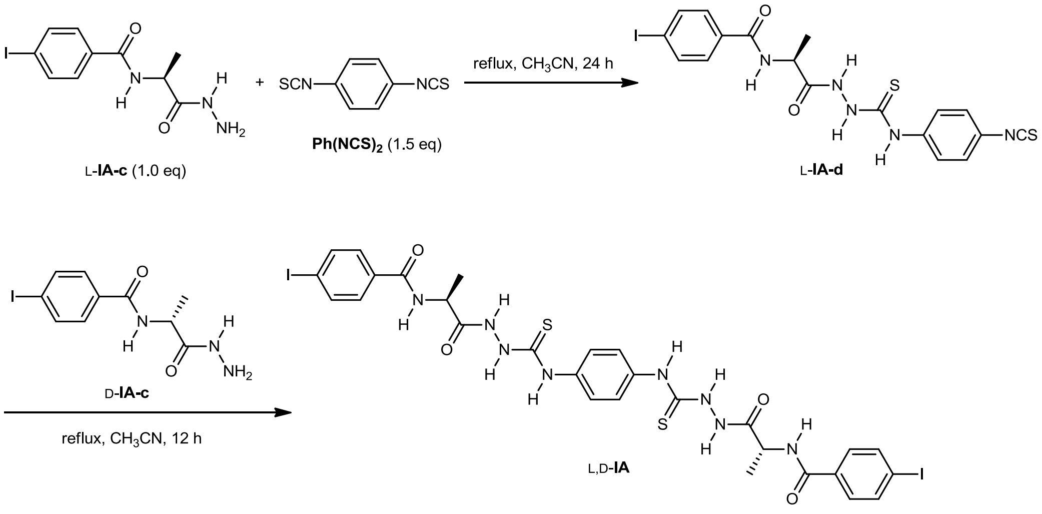

Supplementary Figure 2. General procedures for the syntheses of L,D-**IA**

X-ray crystal structures

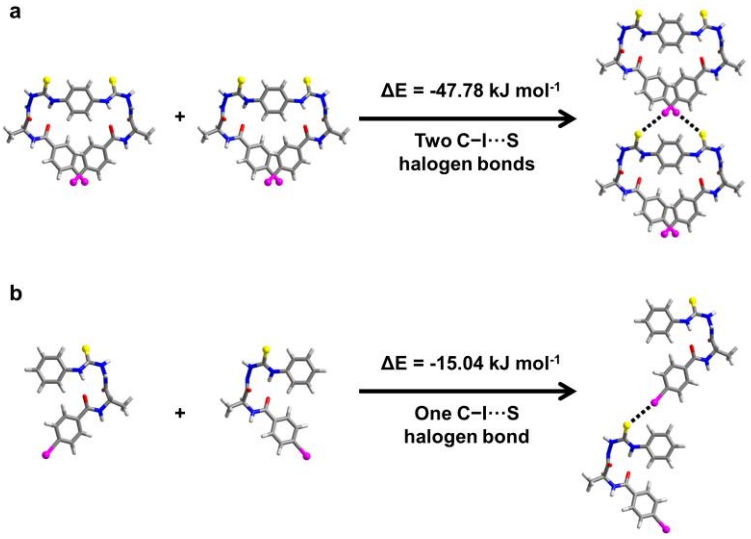

Supplementary Figure 3. Structural illustration for the calculations of the bonding energies in the dimer of bilateral *cis*-L,L-**IA** with two C−I···S halogen bonds (a) and unilateral analogues with only one C−I···S halogen bond (b). Method: DFT wB97XD with the 6-31+G(d, p) basis set for C, H, O, N and S atoms, and LANL2DZ for I atom. The positions of H-atoms were optimized whereas those of other heavy atoms are fixed and taken from the crystal structures.

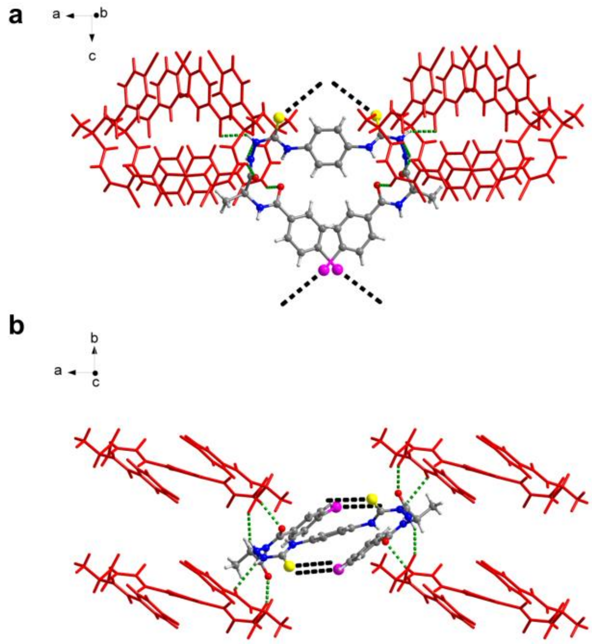

Supplementary Figure 4. Intermolecular hydrogen bonding between one L,L-**IA** molecule with surrounding four molecules as viewed along *b*-axis (a) and *c*-axis (b). Dashed green lines highlight the intermolecular hydrogen bonds, while dashed black lines represent the C−I···S halogen bonds.

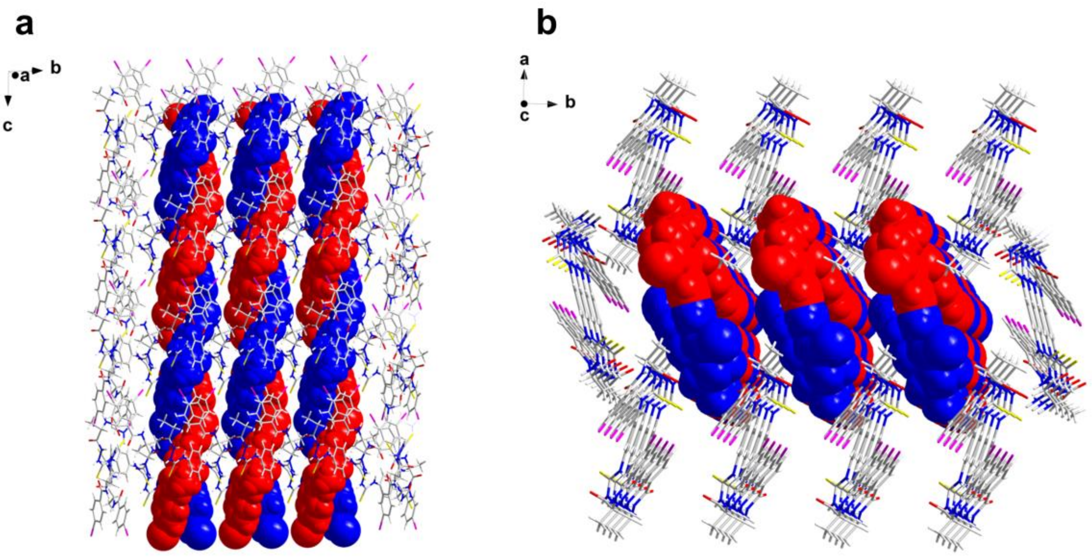

Supplementary Figure 5. 3D supramolecular architectures built from *cis*-form L,L-**IA** as viewed along *a*-axis (a) and *c*-axis (b).

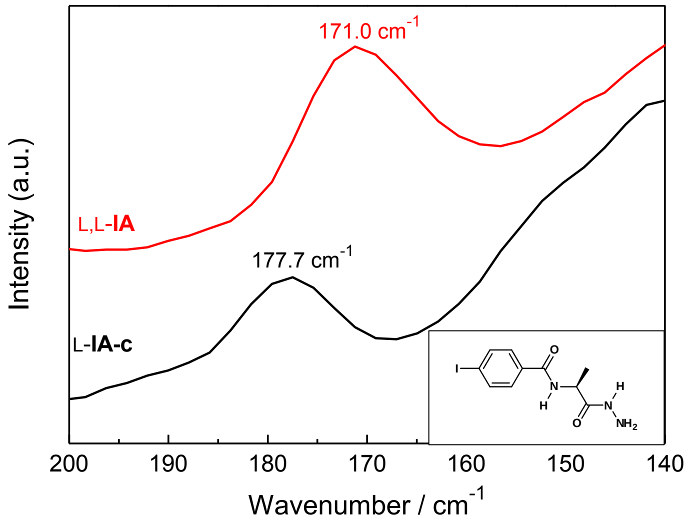

Supplementary Figure 6. Raman spectra of L-**IA-c** and L,L-**IA** in the solid state. Inset is the chemical structure of L-**IA-c** that has not the thiourea moiety, therefore no possibility of the intermolecular C−I···S halogen bonding with this molecule.

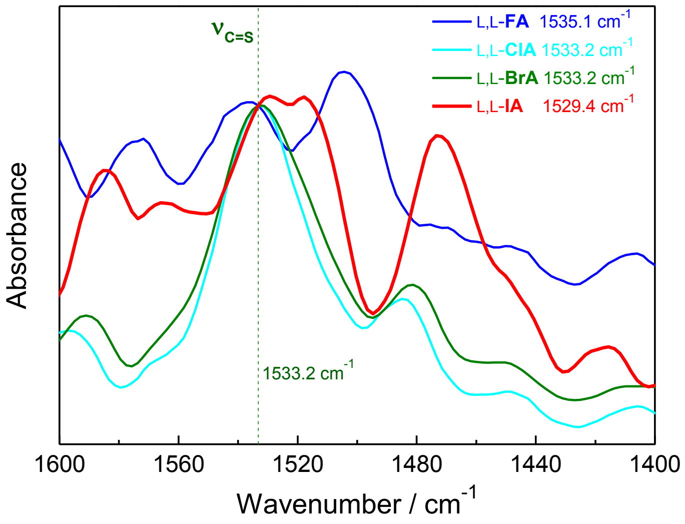

Supplementary Figure 7. Infrared spectra (KBr disk) of L,L-**FA**, L,L-**ClA**, L,L-**BrA** and L,L-**IA** in the solid state.

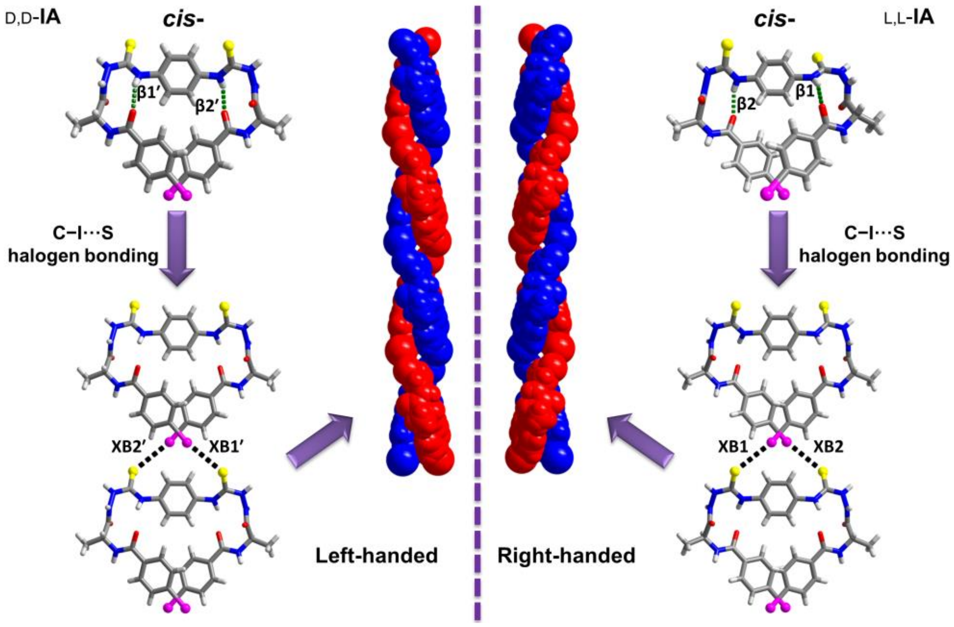

Supplementary Figure 8. Mirror symmetric left-handed and right-handed double helices built from *cis*-form D,D-**IA** and L,L-**IA** through double crossed C−I···S halogen bonding, respectively. Dashed green lines highlight the intramolecular hydrogen bonds represent the β-turn structures while dashed black lines highlight the C−I···S halogen bonds.

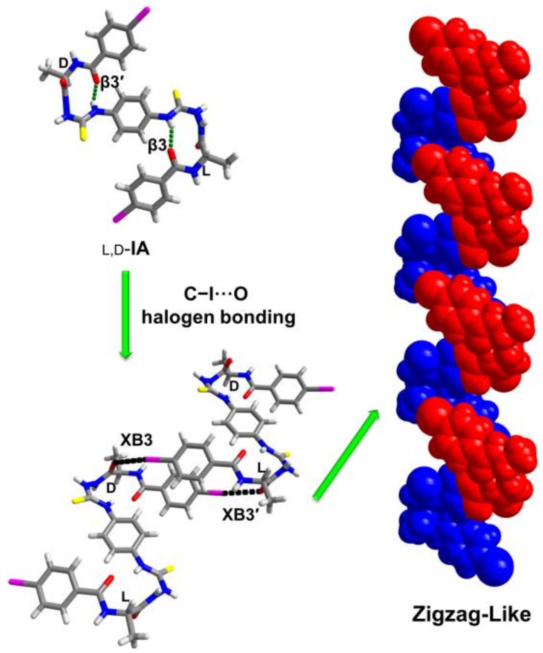

Supplementary Figure 9. Zigzag-like supramolecular structure formed from *trans*-form L,D-**IA** as revealed by the crystal structure. Dashed green lines represent the intramolecular hydrogen bonds that indicating the β-turn structures while dashed black lines highlight the C−I···O halogen bonds. The side contains L-alanine residue is depicted in blue, while D side is in red.

Spectral studies and microscopy images

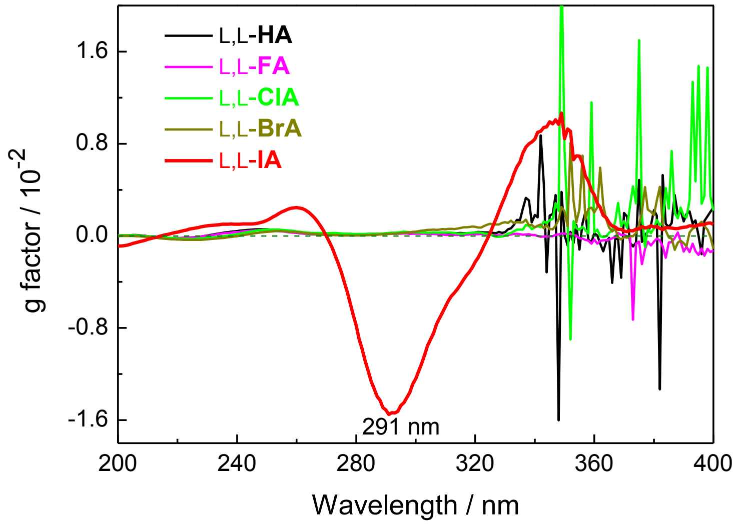

Supplementary Figure 10. The anisotropy factors *g* of L,L-**XA** (**X** = **H**, **F**, **Cl**, **Br**, **I**) in CH_3CN at 25 ℃.

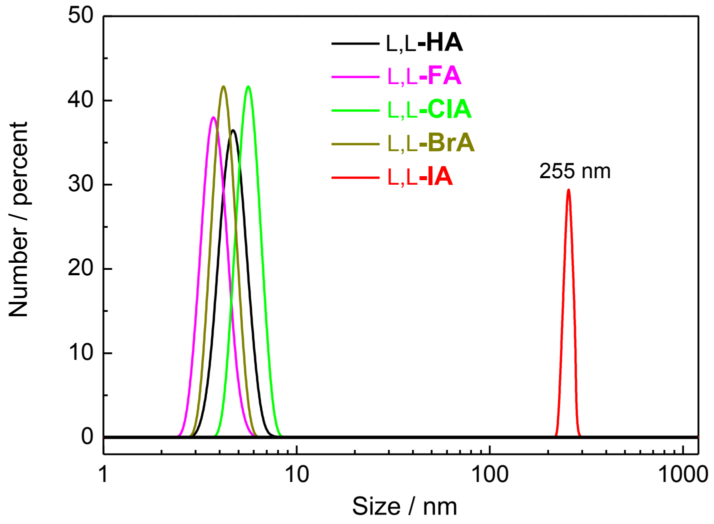

Supplementary Figure 11. Hydrodynamic diameters of L,L-**XA** (**X** = **H**, **F**, **Cl**, **Br**, **I**) in CH_3CN measured by dynamic light scattering at 25 ℃. [L,L-**XA**] = 5 μM. The CH_3CN solutions of L,L-**IA** were prepared through sufficient ultrasonic oscillation and annealing at 75 ℃, resulting in a relatively narrow size distribution with a particle dispersion index 0.17 at the diameters around 255 nm measured from the DLS experiments.

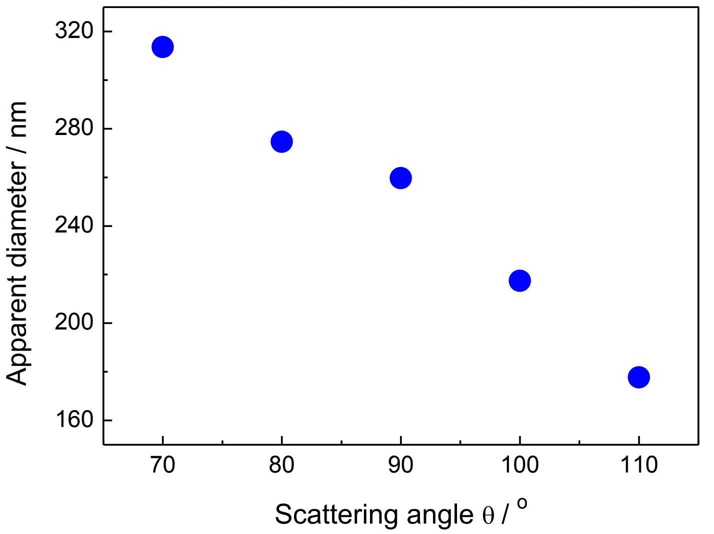

Supplementary Figure 12. Apparent hydrodynamic diameters of L,L-**IA** in CH_3CN measured at different scattering angles θ at 25 ºC. The significant dependence of the hydrodynamic diameters on the scattering angle suggests a pronounced anisotropy of the polymeric species in solution phase. For species of isotropic or quasi-isotropic shape, such as spherical or quasi-spherical clusters, the diameters are invariant or weakly fluctuant at different scattering angles.[1] The conventional hydrodynamic diameters are measured at a fixed angle of 90 °. [L,L-**IA**] = 5 μM.

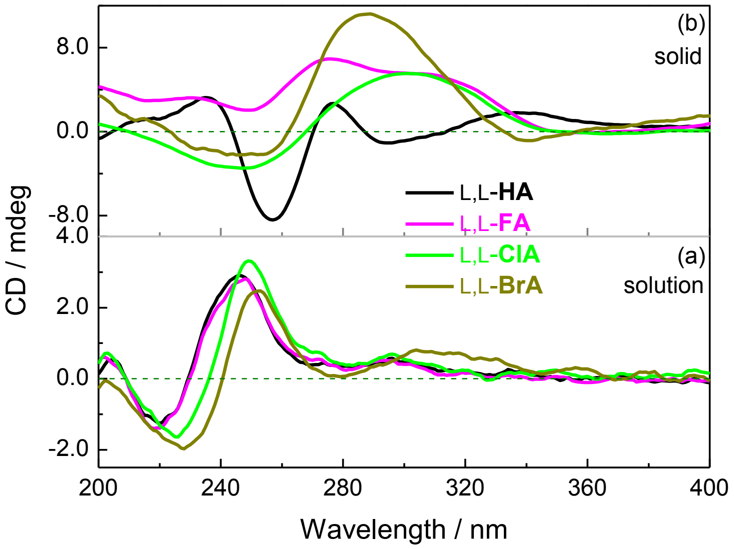

Supplementary Figure 13. CD spectra of L,L-**XA** (**X** = **H**, **F**, **Cl**, **Br**) in CH_3CN solutions at 25 ℃ (a) and in the solid state (b). [L,L-**XA**] = 5 μM in CH_3CN. The concentration of the solid CD sample is 1.0 mg/400 mg KCl.

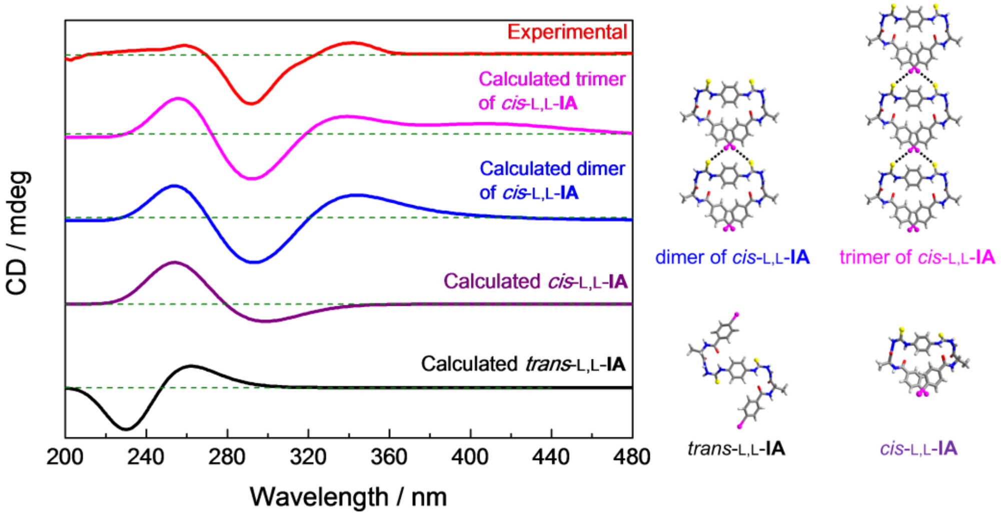

Supplementary Figure 14. The experimental and calculated CD spectra of monomer and oligomers of L,L-**IA** in CH_3CN solutions. The structures for calculations are shown in the right side. Calculation method: TDDFT wB97XD with the 6-31G* basis set for C, H, O, N and S atoms, LANL2DZ for I atoms.

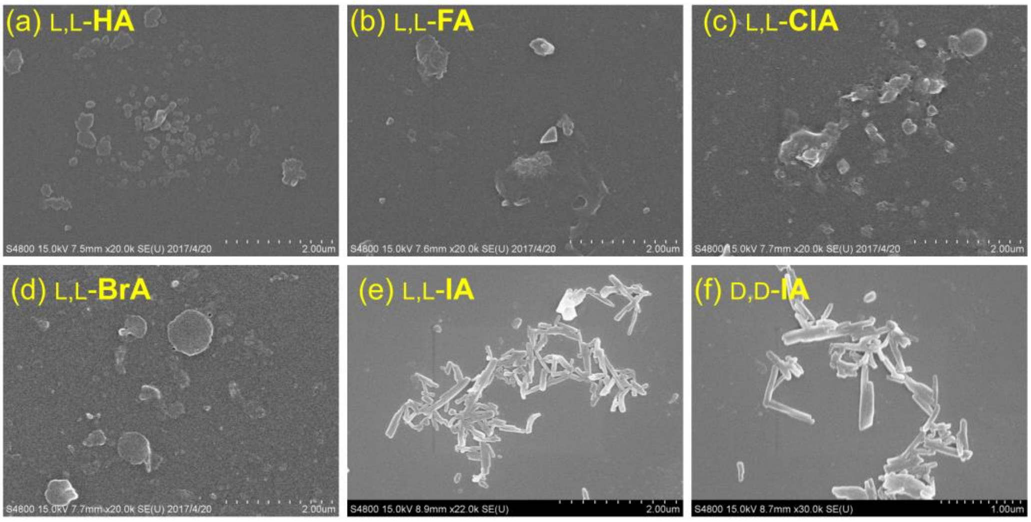

Supplementary Figure 15. SEM images of air-dried samples from CH_3CN solutions (5 μM) of L,L-**HA** (a), L,L-**FA** (b), L,L-**ClA**(c) and L,L-**BrA** (d), L,L-**IA** (e) and D,D-**IA** (f) on platinum coated silicon wafers.

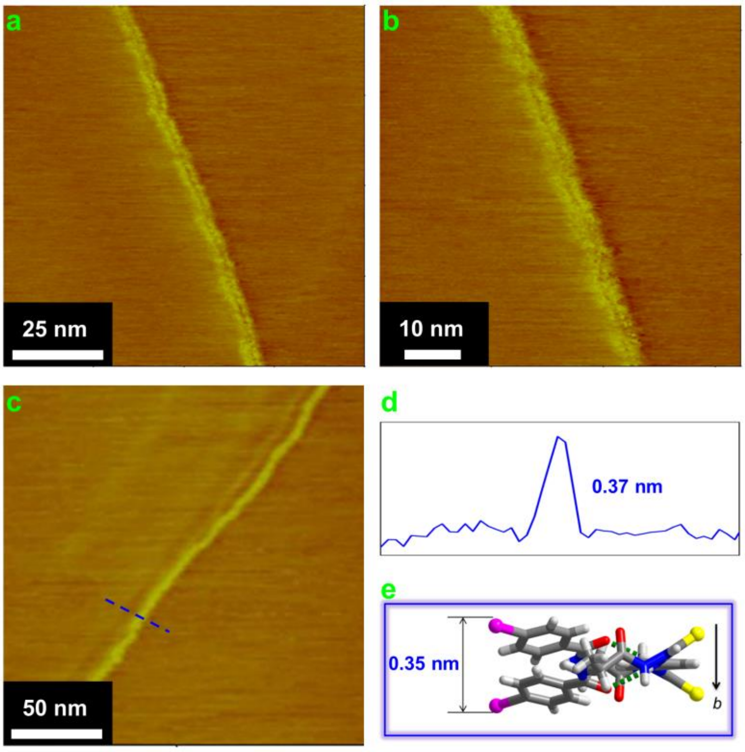

Supplementary Figure 16. STM height images of L,L-**IA** (a, b) and D,D-**IA** (c) at the CH_3CN-highly oriented pyrolytic graphite (HOPG) interface. (d) The height profile along the blue dashed line in (c). (e) The thickness of one *cis*-D,D-**IA** molecule along *b*-axis according to the crystal structures. The CH_3CN solutions for STM experiments were diluted into 1 μM.

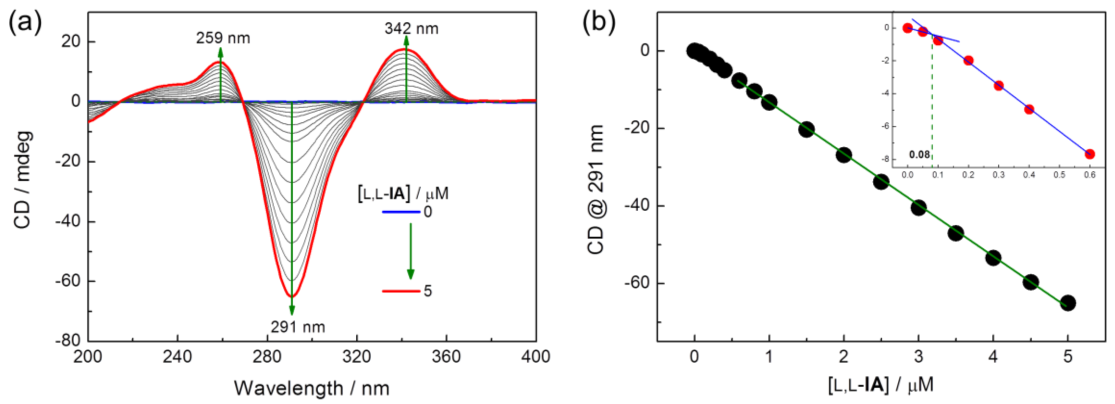

Supplementary Figure 17. (a) Concentration-dependent CD spectra of L,L-**IA** in CH_3CN at 25 ℃. [L,L-**IA**] = 0 - 5 μM. (b) Plots of CD signals at 291 nm versus the concentration of L,L-**IA**. A critical aggregation concentration of L,L-**IA** in CH_3CN, *ca*. 0.08 μM, was deduced from the inflection point in the inset.

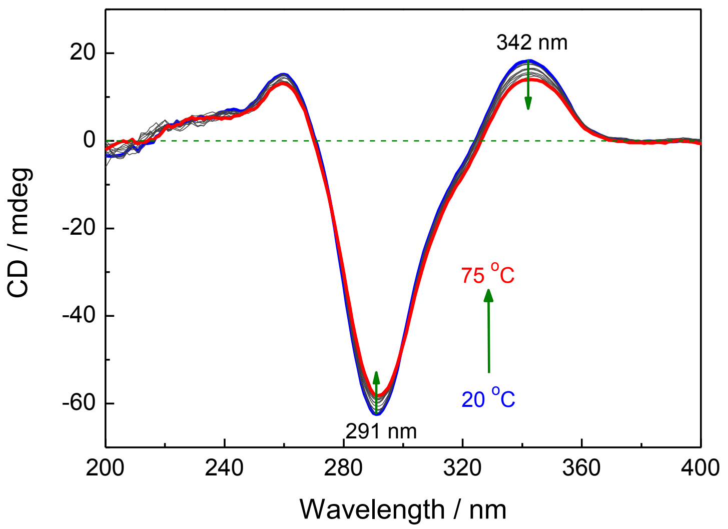

Supplementary Figure 18. Temperature-dependent (20 to 75 ℃) CD spectra of L,L-**IA** in CH_3CN. [L,L-**IA**] = 5 μM.

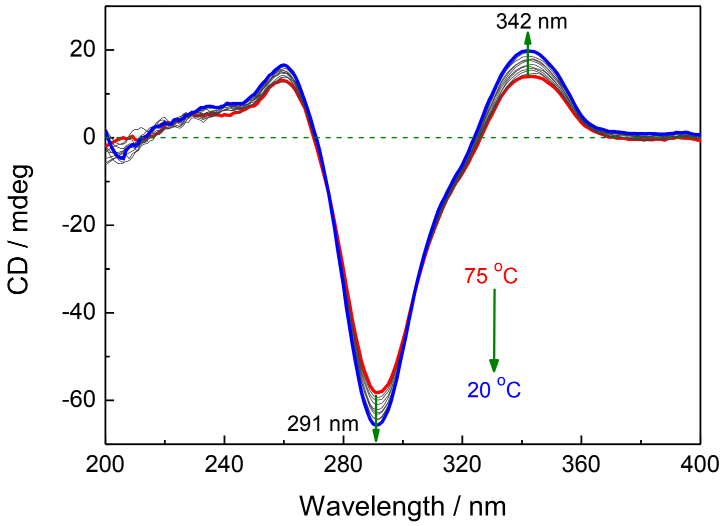

Supplementary Figure 19. Temperature-dependent (75 to 20 ℃) CD spectra of L,L-**IA** in CH_3CN. [L,L-**IA**] = 5 μM.

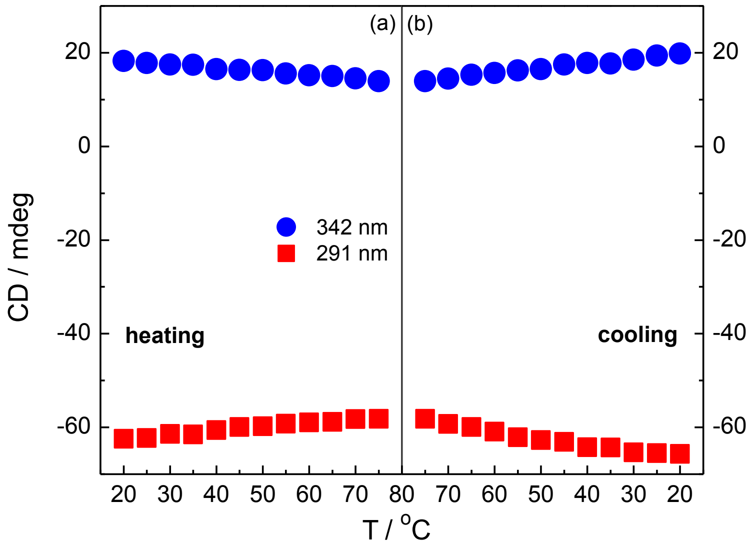

Supplementary Figure 20. Plots of CD intensities at 291 and 342 nm versus solution temperature in the heating (a) and cooling (b) processes. [L,L-**IA**] = 5 μM.

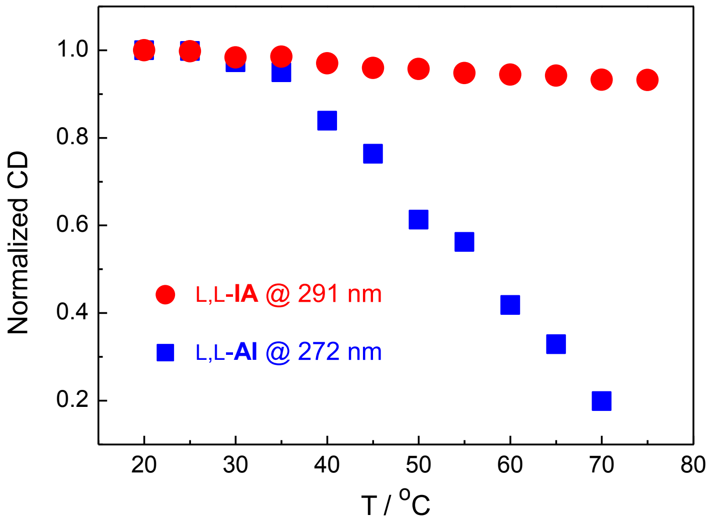

Supplementary Figure 21. Normalized CD intensities at 272 nm of L,L-**AI**[2] and at 291 nm of L,L-**IA** versus CH_3CN solution temperature. [L,L-**AI**] = 20 μM, [L,L-**IA**] = 5 μM.

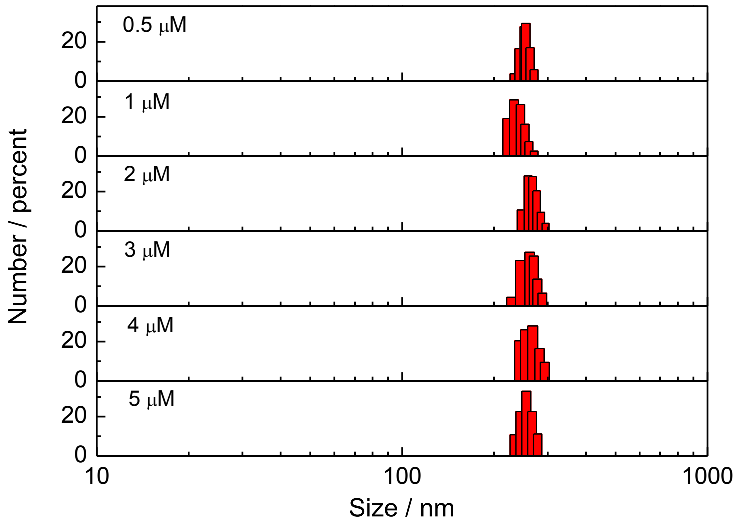

Supplementary Figure 22. Concentration-dependent (0.5 to 5 μM) hydrodynamic diameters of L,L-**IA** in CH_3CN measured by dynamic light scattering at 25 ℃. Measurements at concentrations lower than 0.5 μM were not possible due to the limit of the DLS sensitivity.

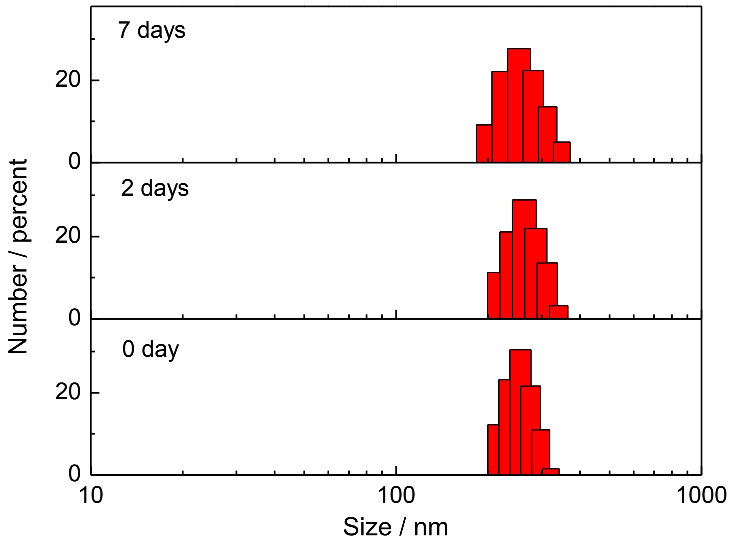

Supplementary Figure 23. Time-dependent (0-7 days) hydrodynamic diameters of L,L-**IA** in CH_3CN measured by dynamic light scattering at 25 ℃. [L,L-**IA**] = 5 μM.

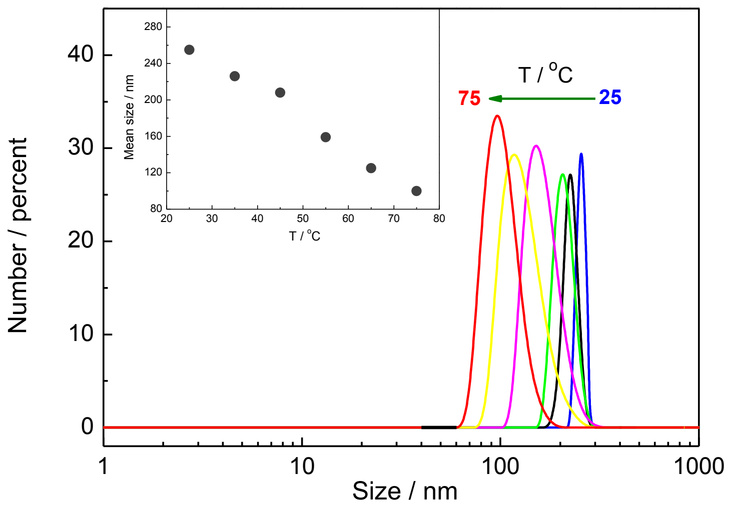

Supplementary Figure 24. Temperature-dependent (25 to 75 ℃) hydrodynamic diameters of L,L-**IA** in CH_3CN measured by dynamic light scattering. Inset is the mean size versus temperature. [L,L-**IA**] = 5 μM.

Spectral responses to halogen anions

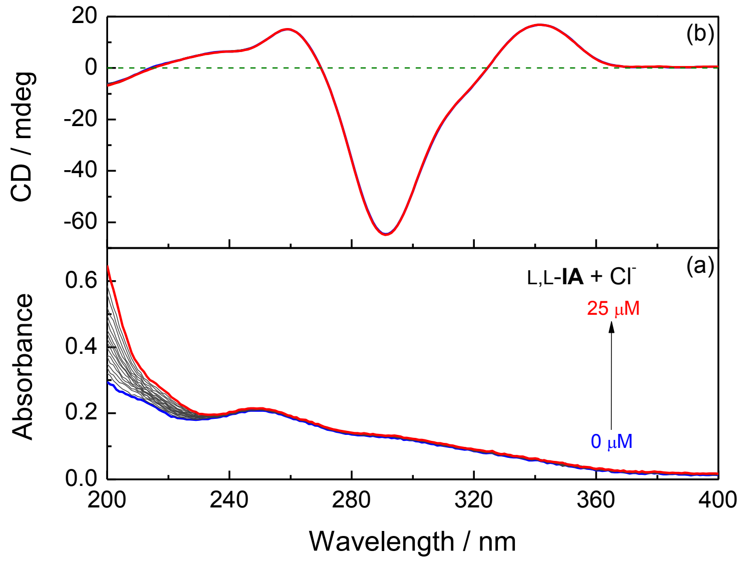

Supplementary Figure 25. Absorption (a) and CD (b) spectra of L,L-**IA** in CH_3CN in the presence of Cl^-. [L,L-**IA**] = 5 μM, [Cl^-] = 0 to 25 μM. Cl^- exists as the $(n\text{-Bu})_4N^+$ salt.

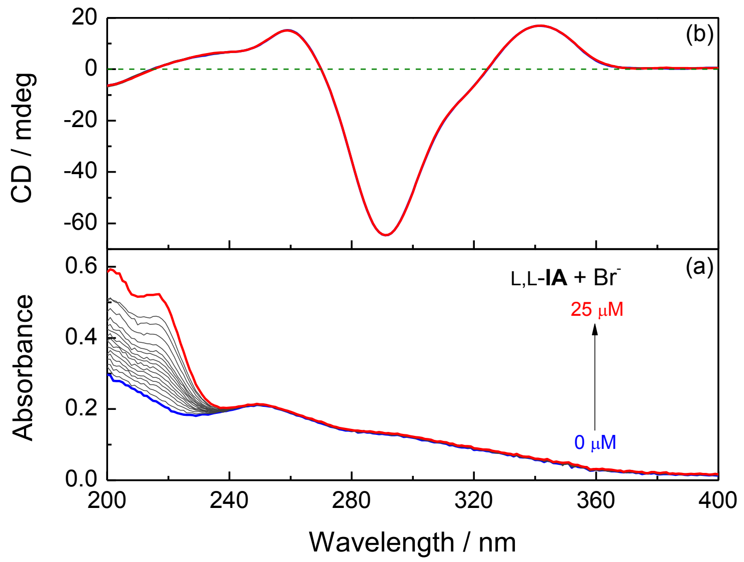

Supplementary Figure 26. Absorption (a) and CD (b) spectra of L,L-**IA** in CH_3CN in the presence of Br^-. [L,L-**IA**] = 5 μM, [Br^-] = 0 to 25 μM. Br^- exists as the $(n\text{-Bu})_4N^+$ salt.

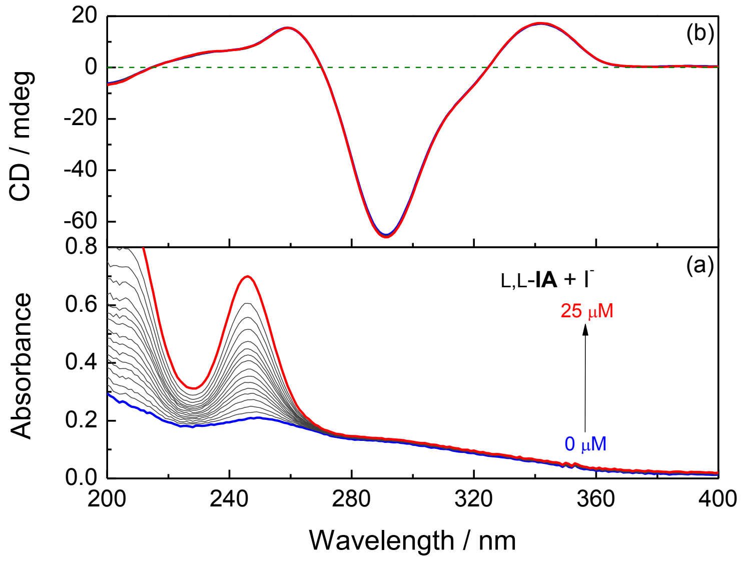

Supplementary Figure 27. Absorption (a) and CD (b) spectra of L,L-**IA** in CH_3CN in the presence of I^-. [L,L-**IA**] = 5 μM, [I^-] = 0 to 25 μM. I^- exists as the (n-Bu)$_4N^+$ salt.

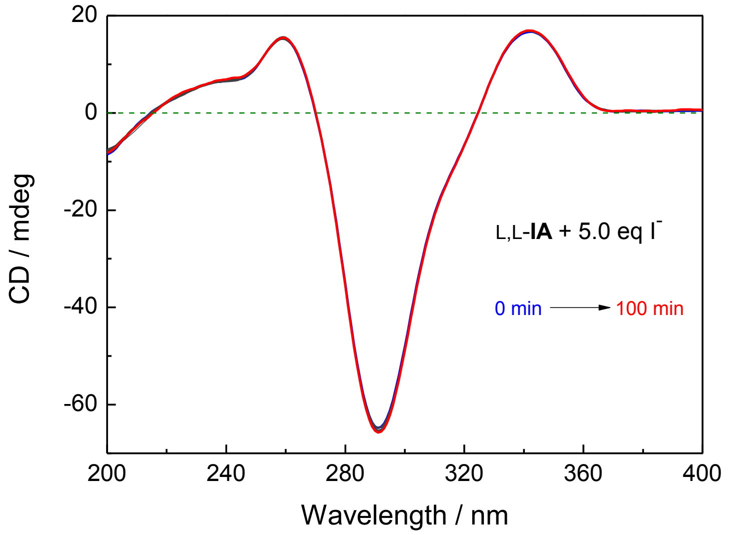

Supplementary Figure 28. Time-dependent CD spectra of L,L-**IA** in the presence of 5 eq I^- in CH_3CN. [L,L-**IA**] = 5 μM, [I^-] = 25 μM. I^- exists as the (n-Bu)$_4N^+$ salt.

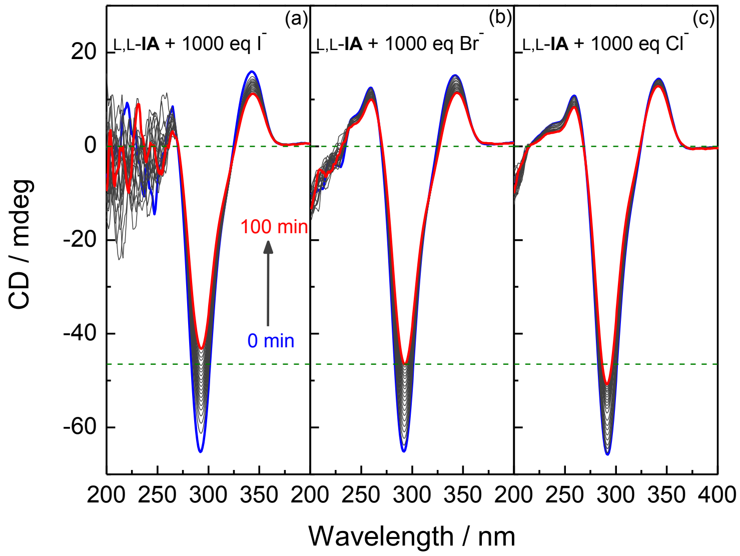

Supplementary Figure 29. Time-dependent CD spectra of L,L-**IA** in the presence of 1000 eq I^- (a), Br^- (b) and Cl^- (c) in CH_3CN. [L,L-**IA**] = 5 μM, [I^-] = [Br^-] = [Cl^-] = 5000 μM. I^-, Br^- and Cl^- exist as the (n-Bu)$_4N^+$ salt.

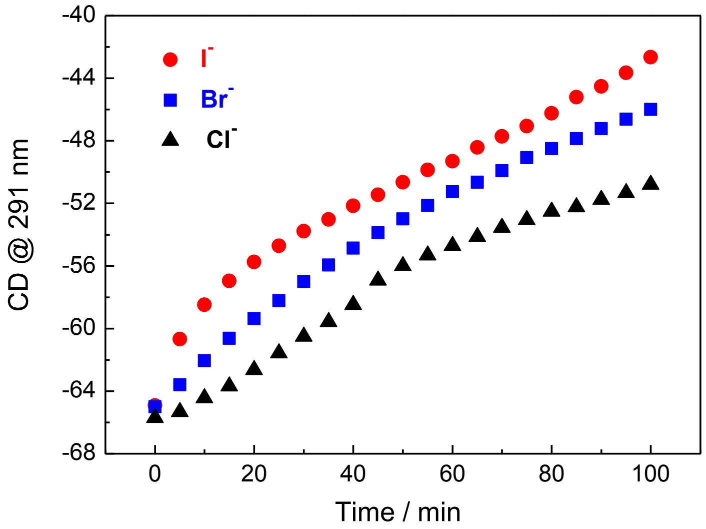

Supplementary Figure 30. Time profiles of CD signals at 291 nm of L,L-**IA** in CH_3CN in the presence of 1000 eq I^-, Br^- and Cl^-. [L,L-**IA**] = 5 μM, [I^-] = [Br^-] = [Cl^-] = 5000 μM. I^-, Br^- and Cl^- exist as the (n-Bu)$_4N^+$ salt.

Influence of solvent

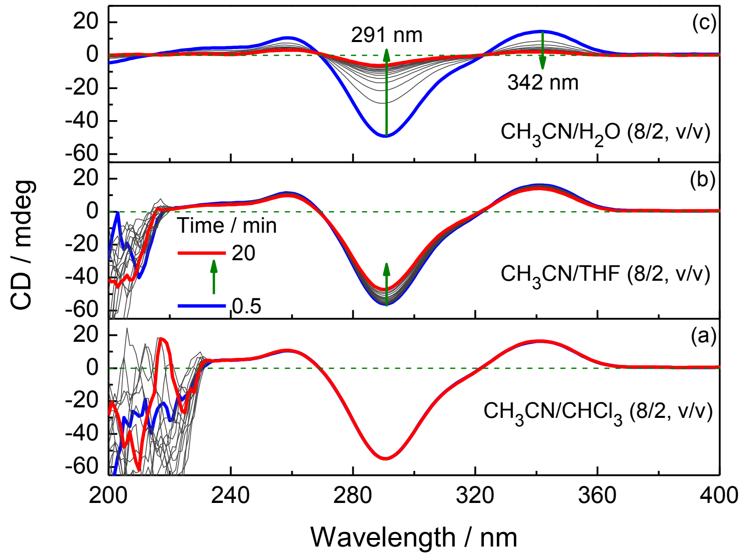

Supplementary Figure 31. Time-dependent CD spectra of L,L-**IA** in CH_3CN with 20% volume fraction of $CHCl_3$ (a), THF (b) and H_2O (c). [L,L-**IA**] = 4 μM.

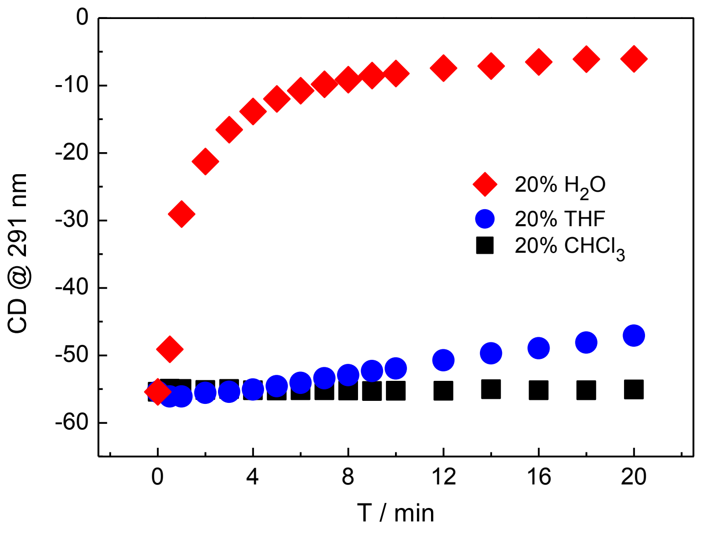

Supplementary Figure 32. Plots versus time of the CD intensity at 291 nm of L,L-**IA** in CH_3CN with 20% volume fraction of $CHCl_3$, THF and H_2O. [L,L-**IA**] = 4 μM.

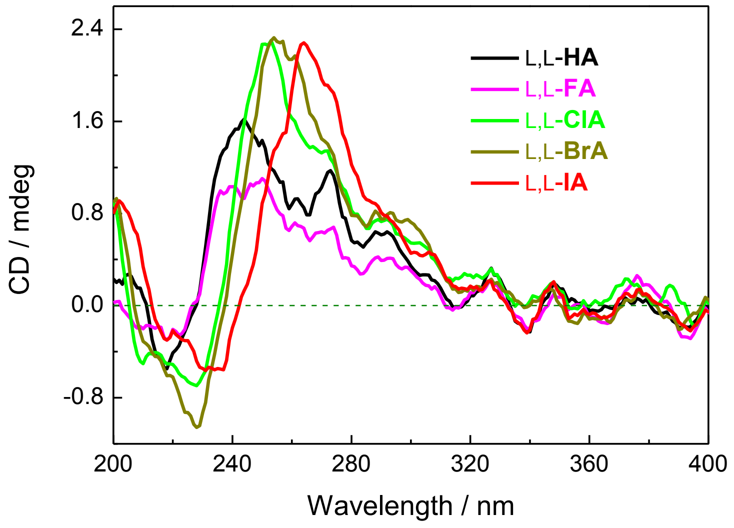

Supplementary Figure 33. CD spectra of L,L-**XA** (**X** = **H**, **F**, **Cl**, **Br**, **I**) in CH_3CN with 20% volume fraction of H_2O after standing for 30 hours. [L,L-**XA**] = 4 μM.

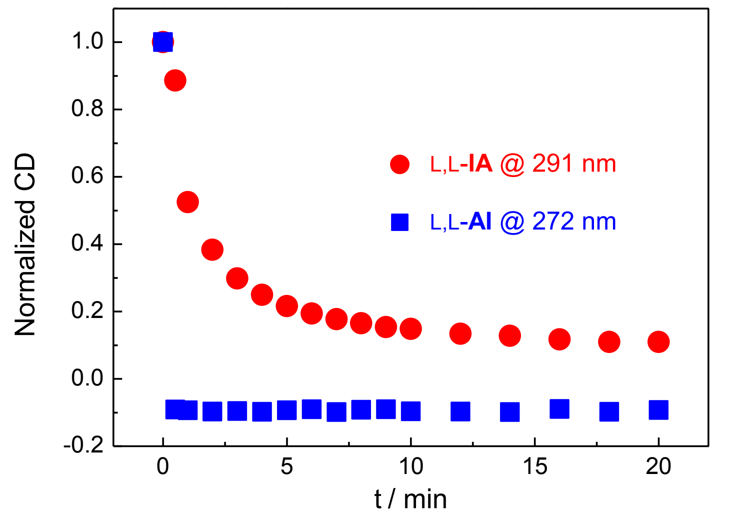

Supplementary Figure 34. Time-dependent normalized CD intensity at 291 nm of L,L-**IA** and 272 nm of L,L-**AI**[2] in CH_3CN with 20% volume fraction of H_2O. [L,L-**AI**] = 16 μM, [L,L-**IA**] = 4 μM.

NMR studies

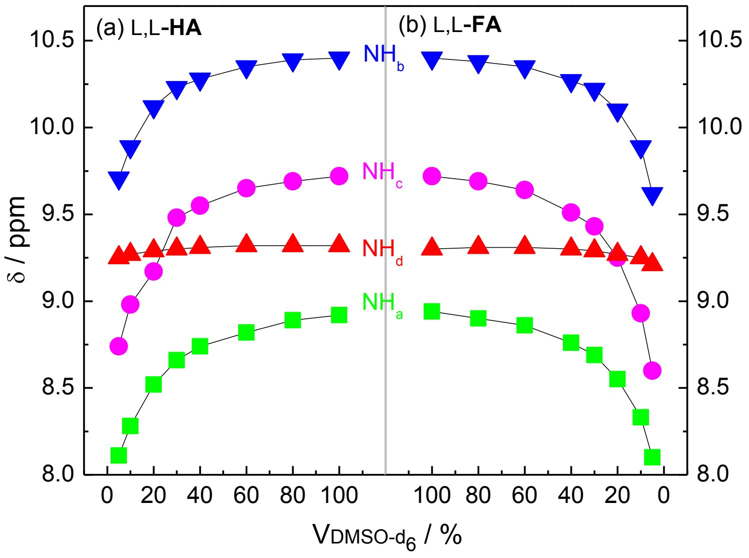

Supplementary Figure 35. Influence on -NH proton resonances of L,L-**HA** (a) and L,L-**FA** (b) in CD_3CN/DMSO-*d*$_6$ mixtures by the volume fraction of DMSO-*d*$_6$ (500 MHz, 25 ℃). [L,L-**HA**] = [L,L-**FA**] = 2 mM.

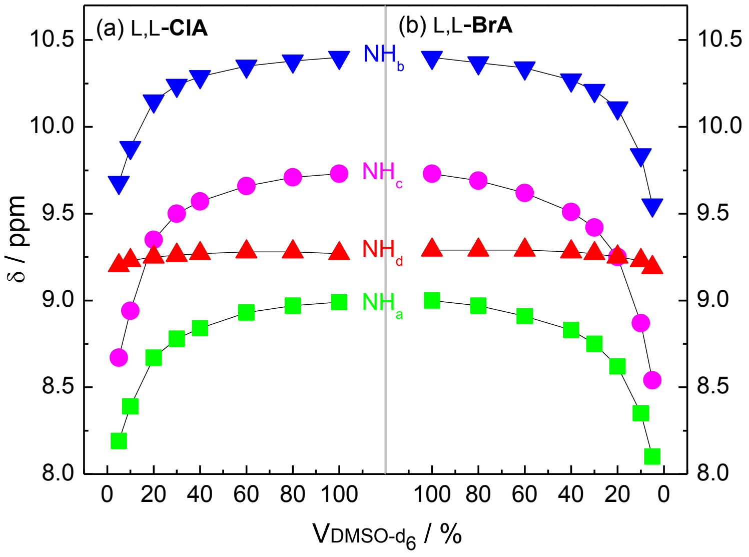

Supplementary Figure 36. Influence on -NH proton resonances of L,L-**ClA** (a) and L,L-**BrA** (b) in CD_3CN/DMSO-*d*$_6$ mixtures by the volume fraction of DMSO-*d*$_6$ (500 MHz, 25 ℃). [L,L-**ClA**] = [L,L-**BrA**] = 2 mM.

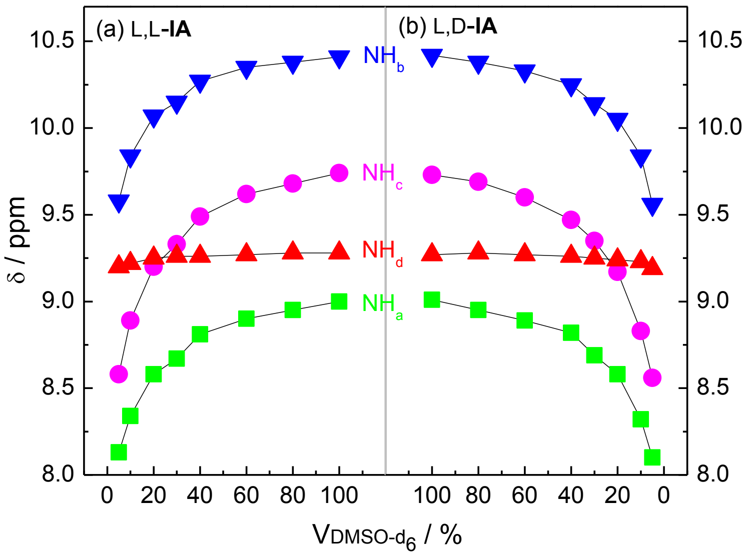

Supplementary Figure 37. Influence on -NH proton resonances of L,L-**IA** (a) and L,D-**IA** (b) in CD_3CN/DMSO-d_6 mixtures by the volume fraction of DMSO-d_6 (500 MHz, 25 ℃). [L,L-**IA**] = [L,D-**IA**] = 2 mM.

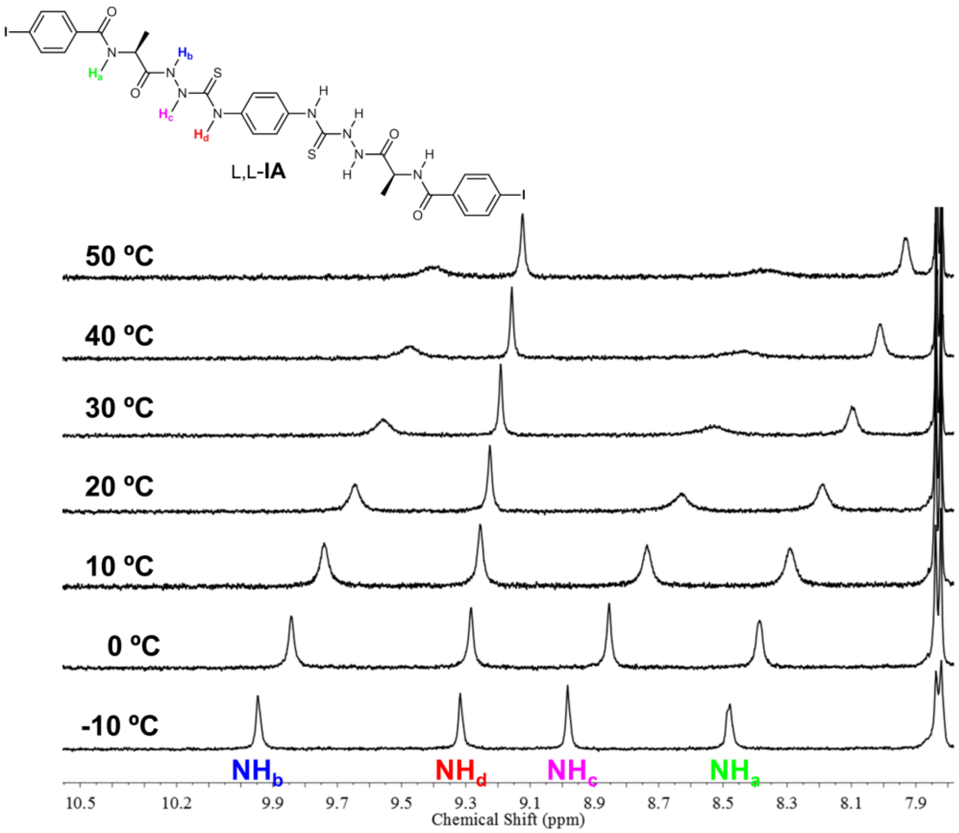

Supplementary Figure 38. Temperature-dependent partial ^{1}H NMR spectra of -NH protons of L,L-**IA** (500 MHz) in 95:5 (v/v) CD_3CN/DMSO-d_6 mixtures. [L,L-**IA**] = 1 mM.

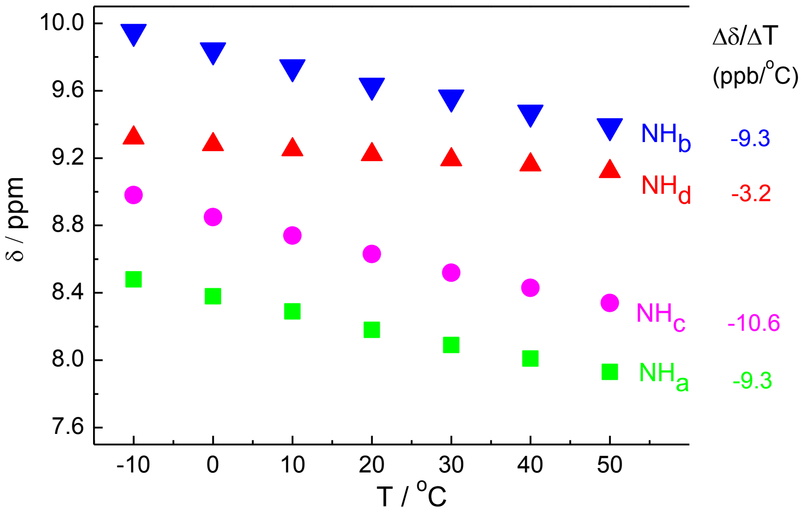

Supplementary Figure 39. Influence on -NH proton resonances of L,L-**IA** in 95:5 (v/v) CD_3CN/DMSO-d_6 mixtures by temperatures (500 MHz) and the fitted temperature coefficients.[3] [L,L-**IA**] = 1 mM.

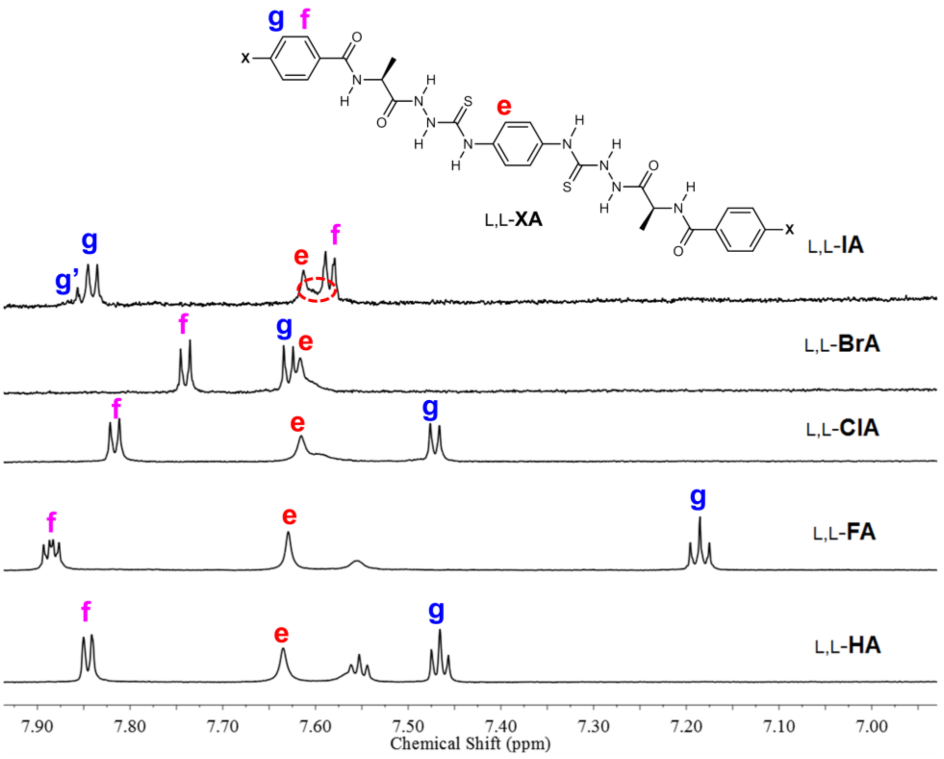

Supplementary Figure 40. Partial 850 MHz ^{1}H NMR spectra of protons on benzene rings (H_e, H_f and H_g) in L,L-**XA** in CD_3CN at 25 ℃. The solutions were samples saturated.

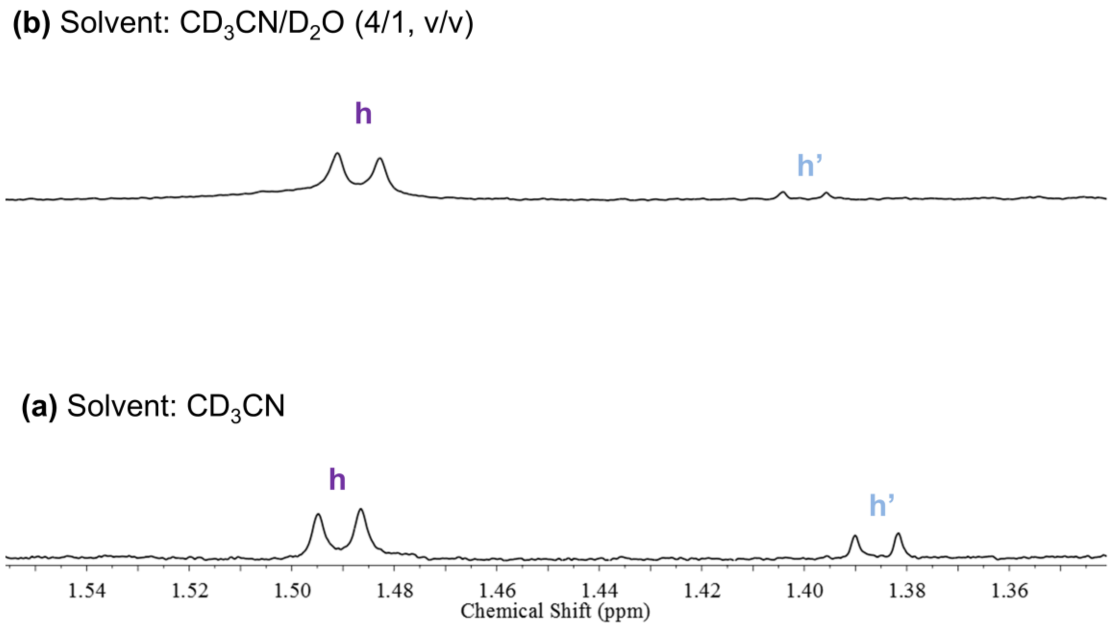

Supplementary Figure 41. Partial 850 MHz ^{1}H NMR spectra of $-CH_{3h}$ of L,L-**IA** in CD_3CN (a) and 4:1 (v/v) CD_3CN/D_2O (b) at 25 ℃. The solutions were samples saturated (*ca*. 5.6 μM).

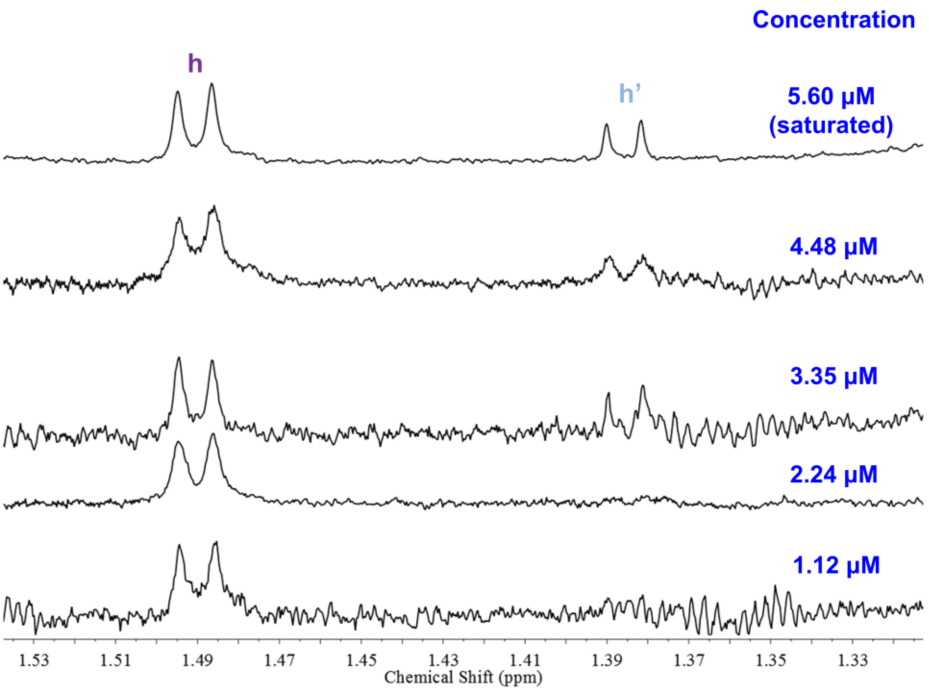

Supplementary Figure 42. Concentration-dependent partial 850 MHz ^{1}H NMR spectra of $-CH_{3h}$ of L,L-**IA** in CD_3CN at 25 ℃. At lower concentration, *i.e.* 1.12 μM, due to the sensitivity limit of the NMR technique, in particular to the oligomeric species, the signals for the helical oligomers of the *cis*-form L,L-**IA** (h') could not be observed.

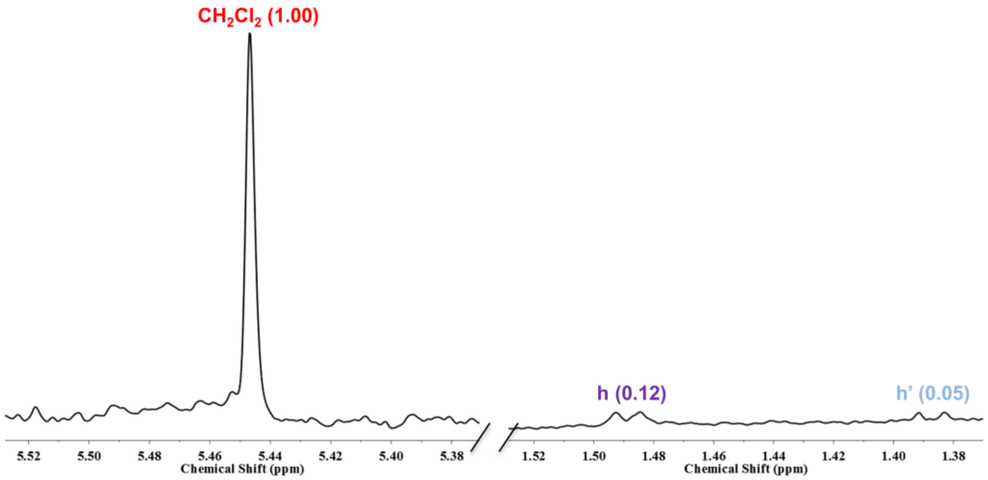

Supplementary Figure 43. Partial 850 MHz ^1H NMR spectrum of $–CH_{3h}$ of L,L-**IA** and CH_2Cl_2 in CD_3CN at 25 ℃. [L,L-**IA**] = 5 μM, [CH_2Cl_2] = 15 μM. CH_2Cl_2 was used as an internal standard. According to the relative integrals given in the parentheses, the percentages of monomers and oligomers are estimated to be 12% and 5%, respectively, whereas that of the remaining, with invisible signals, large polymeric chain structures is 83%.

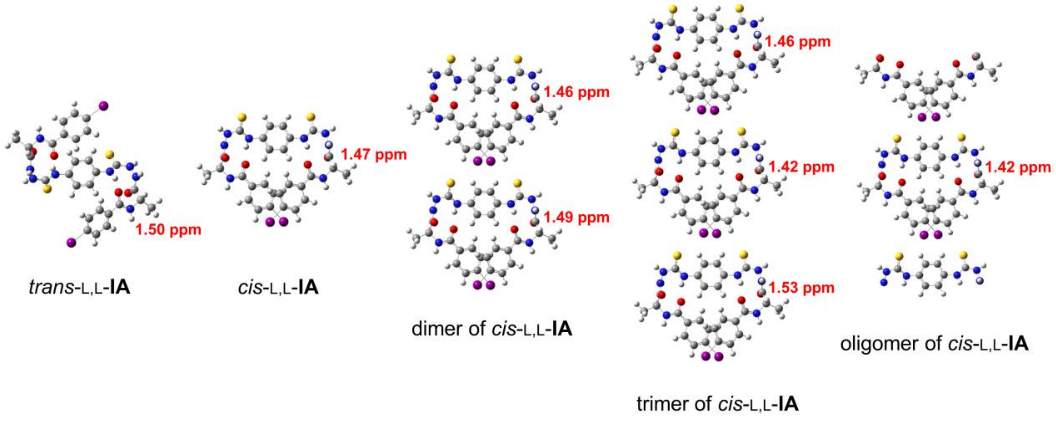

Supplementary Figure 44. Calculated chemical shifts of $-CH_3$ groups in *trans*-form and *cis*-form L,L-**IA** and in dimer, trimer, oligomer of *cis*-form L,L-**IA** in CH_3CN. Method for structural optimization: DFT wB97XD with the 6-31+G(d, p) basis set for C, H, O, N and S atoms, and LANL2DZ for I atom, opt = z-matrix, 0 for H, other atoms are fixed according to crystal structures. Method for NMR calculation: DFT OPBE with the 6-311+G(2d, p) basis set for C, H, O, N and S atoms, and LANL2DZ for I atom.

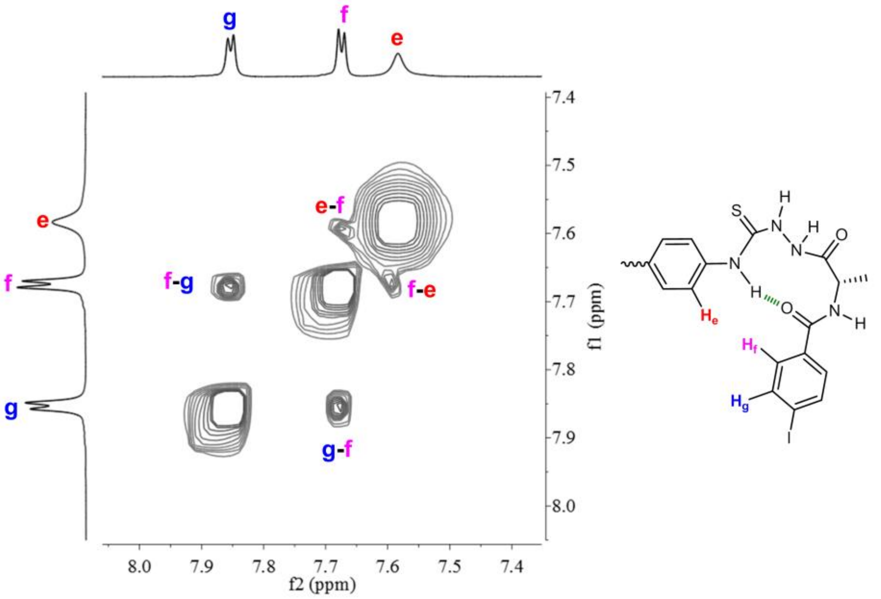

Supplementary Figure 45. Expanded 2D NOESY spectrum (850 MHz, 25 ℃, mixing time 800 ms) of couplings between protons in phenyl rings in L,L-**IA** in DMSO-d_6 in which L,L-**IA** was shown to exist in monomer form. Molecular structure showing β-turn in L,L-**IA** is also presented, with protons labeled. [L,L-**IA**] = 4 mM.

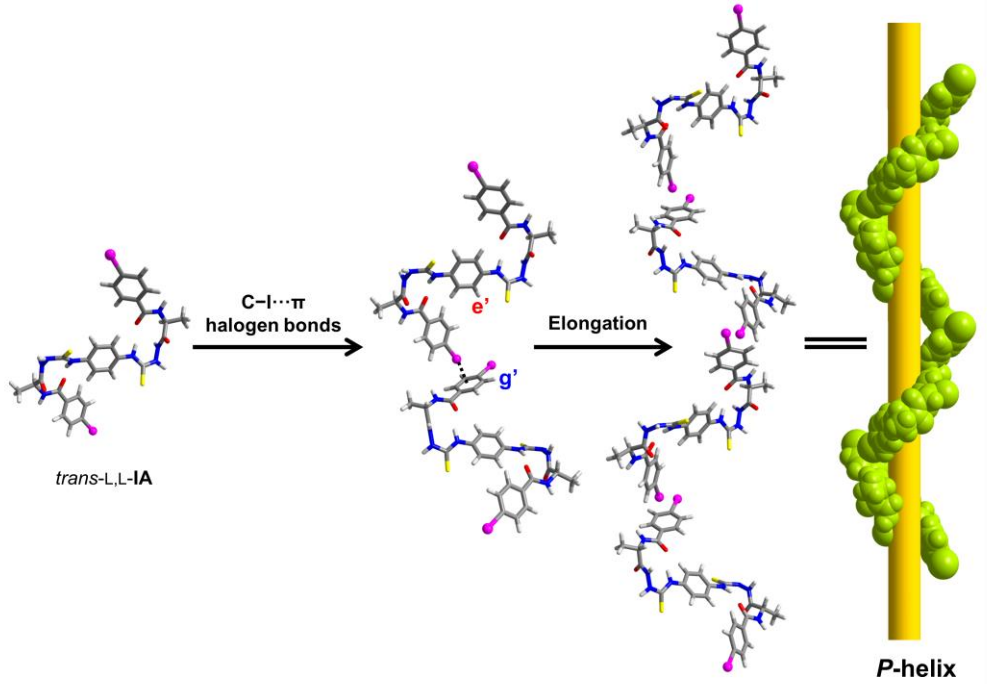

Supplementary Figure 46. Proposed right-handed single-stranded *P*-helix formed from *trans*-form L,L-**IA** molecules as driven by the head-to-tail C−I···π halogen bonds,[2] in which intermolecular $H_{e'}$ and $H_{g'}$ protons are separated by 8.687 Å, that would not lead to intermolecular NOE couplings.

Study of homochiral self-sorting

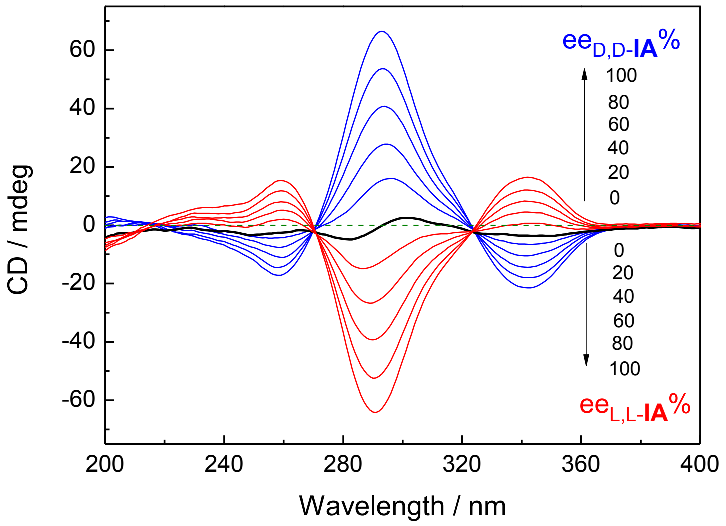

Supplementary Figure 47. CD spectra of **IA** of varying *ee* in CH_3CN at 25 ℃. [L,L-**IA**] + [D,D-**IA**] = 5 μM.

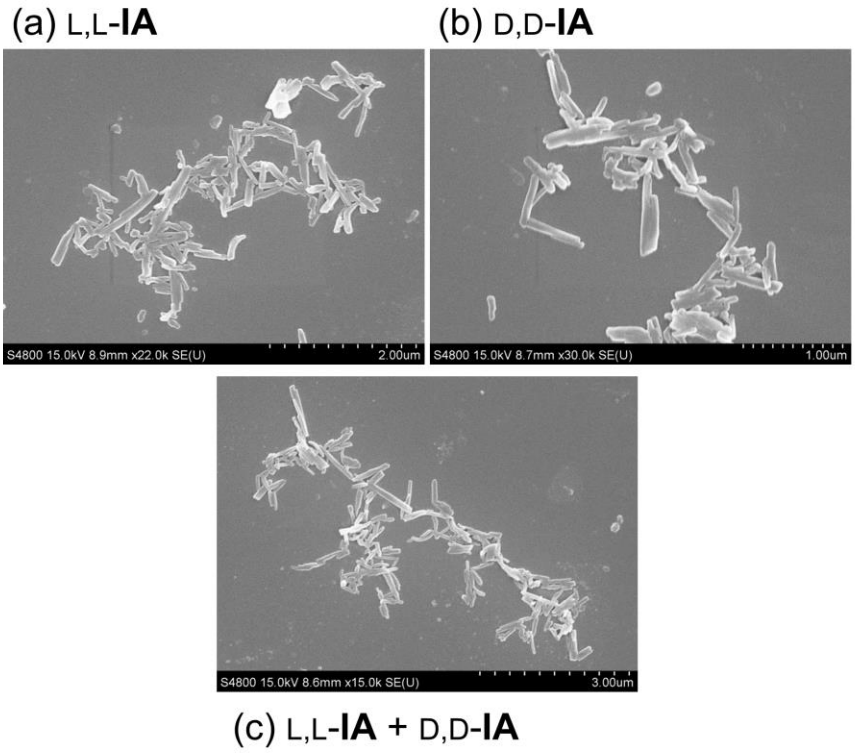

Supplementary Figure 48. SEM images of air-dried samples from CH_3CN solutions of L,L-**IA** (a), D,D-**IA** (b) and equal molar mixture of L,L-**IA** and D,D-**IA** (c) on platinum coated silicon wafers.

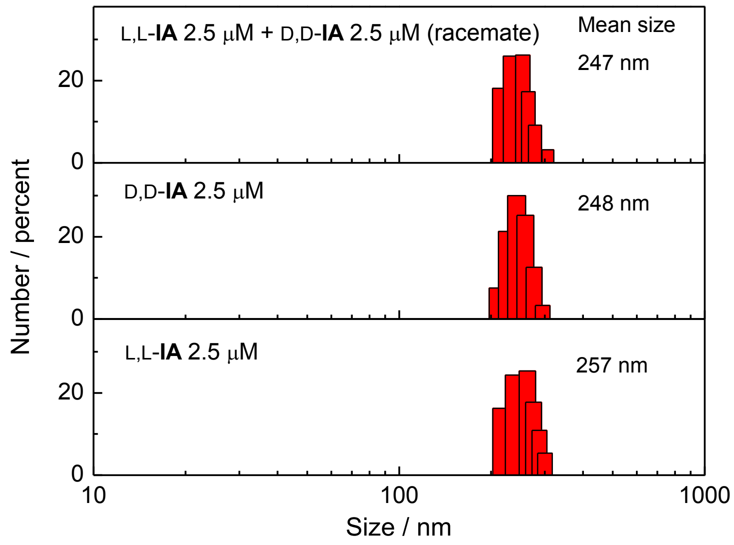

Supplementary Figure 49. Hydrodynamic diameters of L,L-**IA** (a), D,D-**IA** (b) and their racemate (c) in CH_3CN measured by dynamic light scattering at 25 ℃.

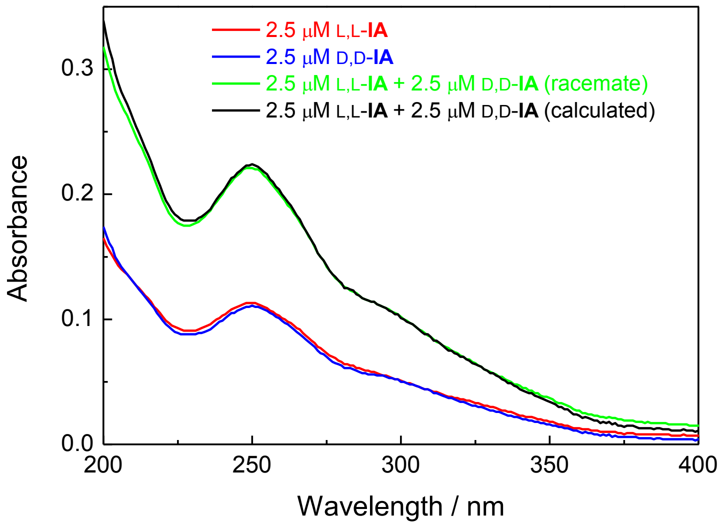

Supplementary Figure 50. Absorption spectra of L,L-**IA**, D,D-**IA** and their racemate in CH_3CN at 25 ℃. The black line is calculated by the addition of the absorption spectra of L,L-**IA** (red line) and D,D-**IA** (blue line), which is practically the same as the experimental spectrum of the racemate (green line).

^{1}H NMR and ^{13}C NMR spectra

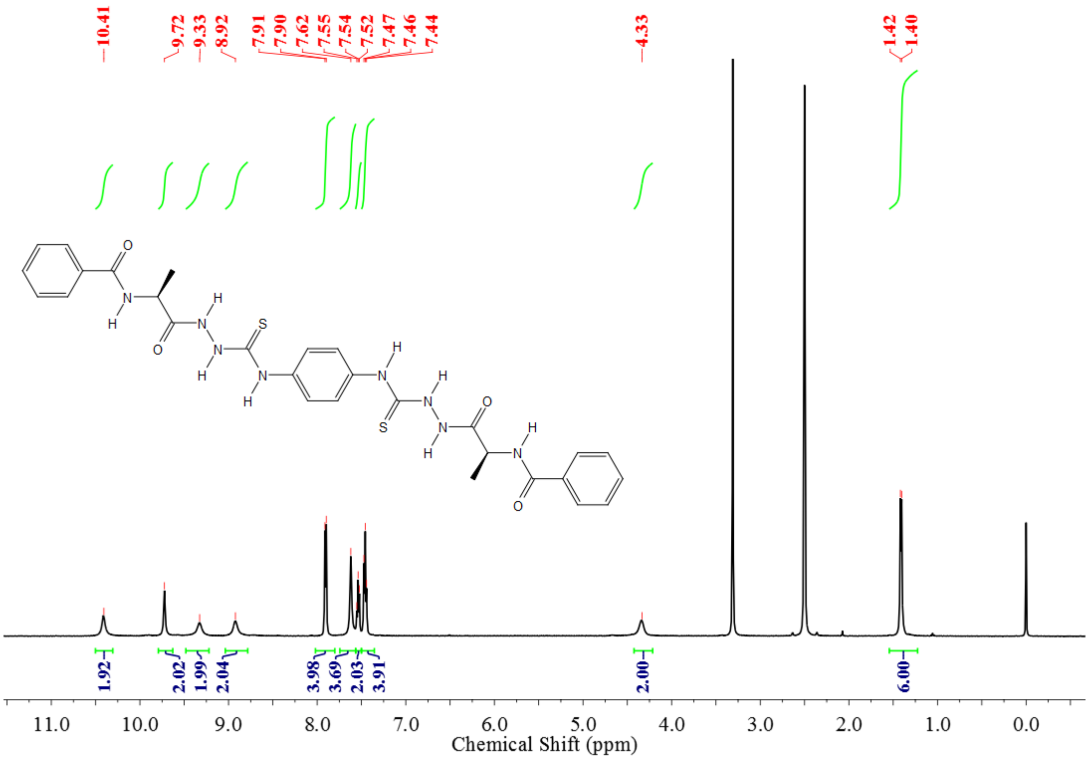

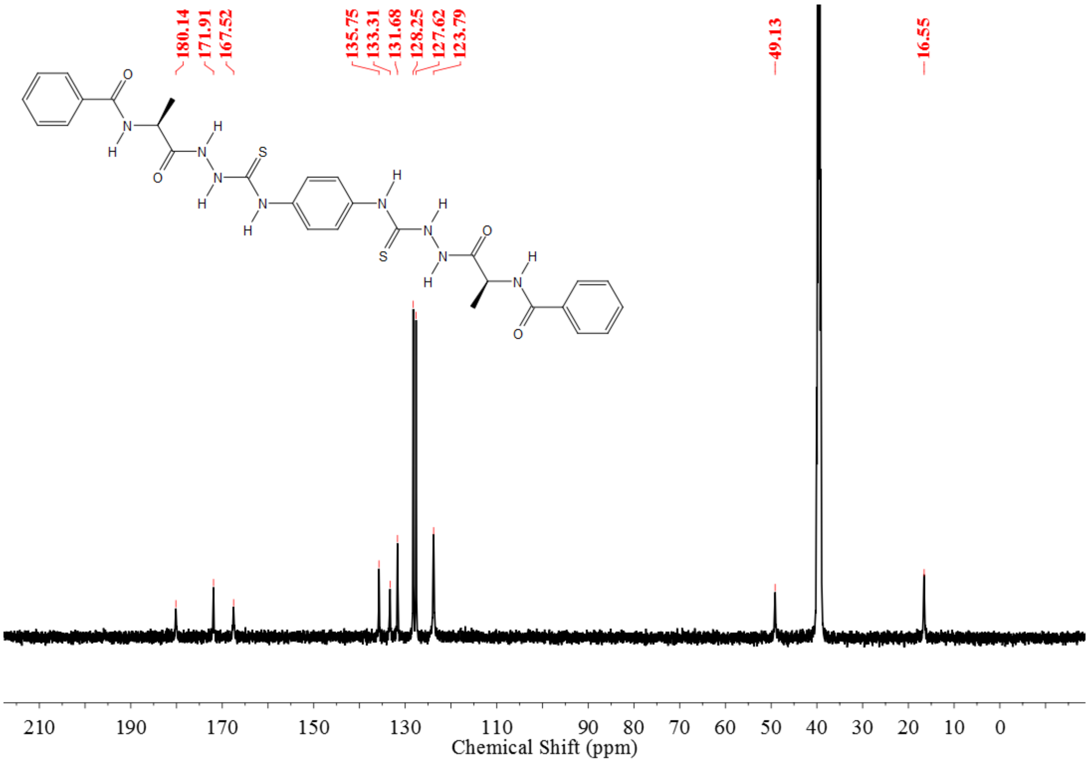

Supplementary Figure 51. ^{1}H and ^{13}C NMR spectra of L,L-**HA**.

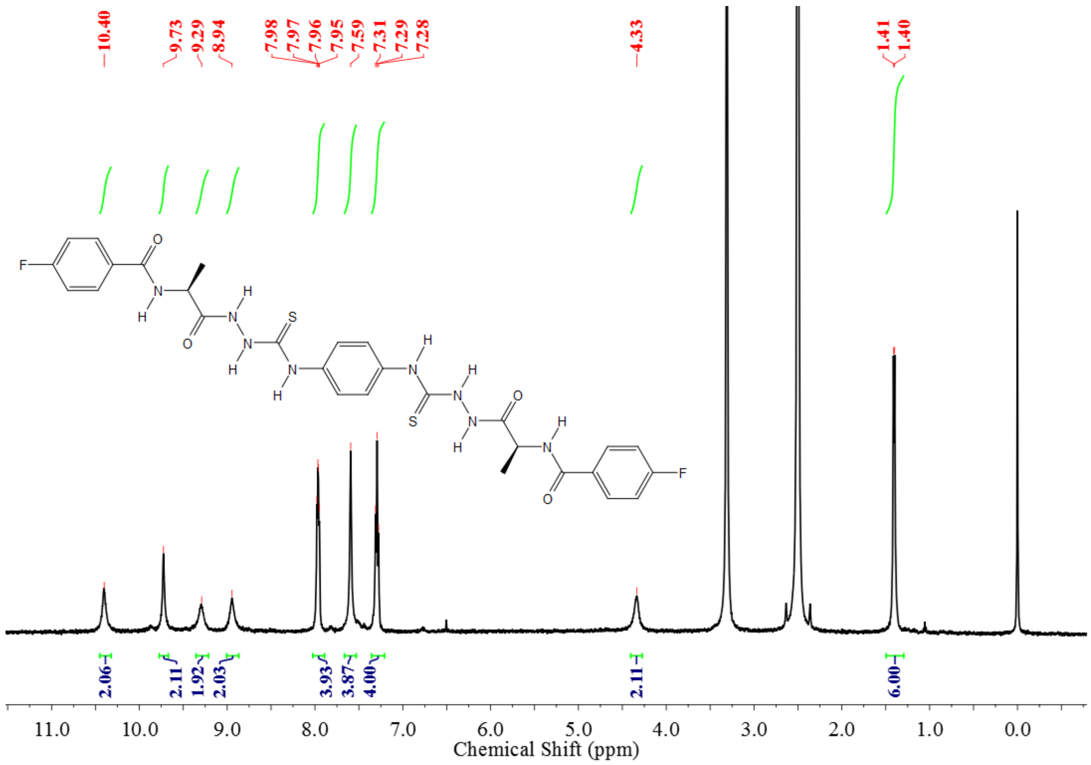

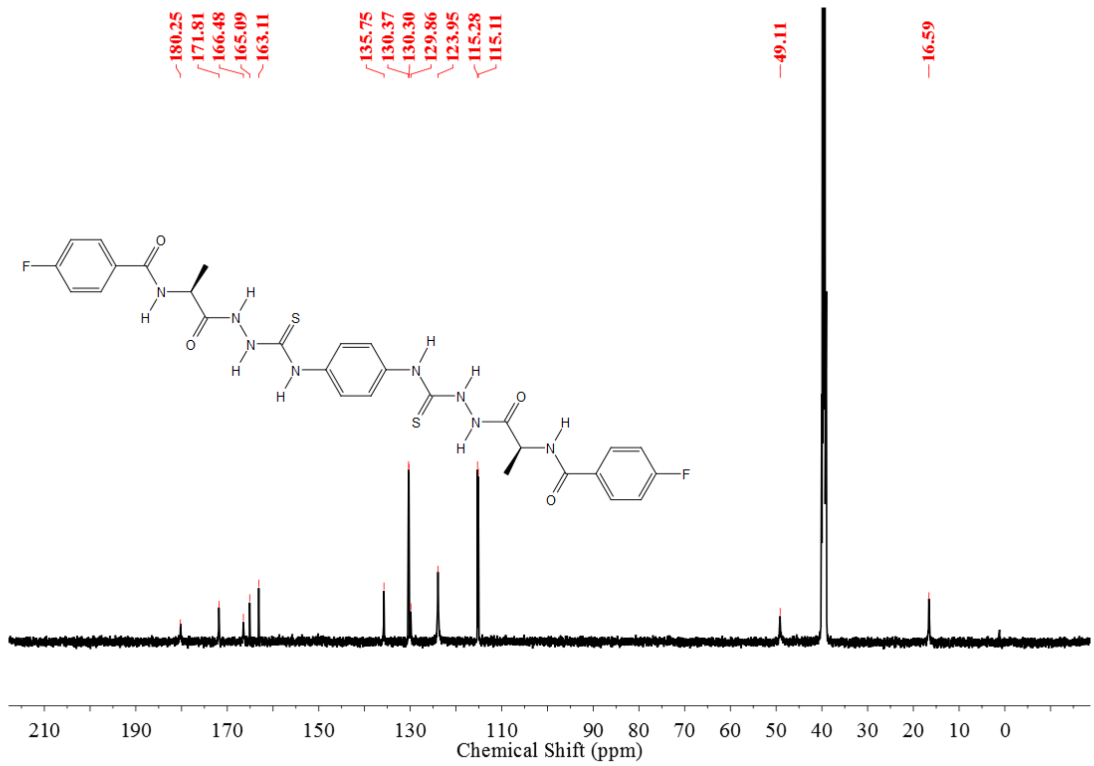

Supplementary Figure 52. ^{1}H and ^{13}C NMR spectra of L,L-**FA**.

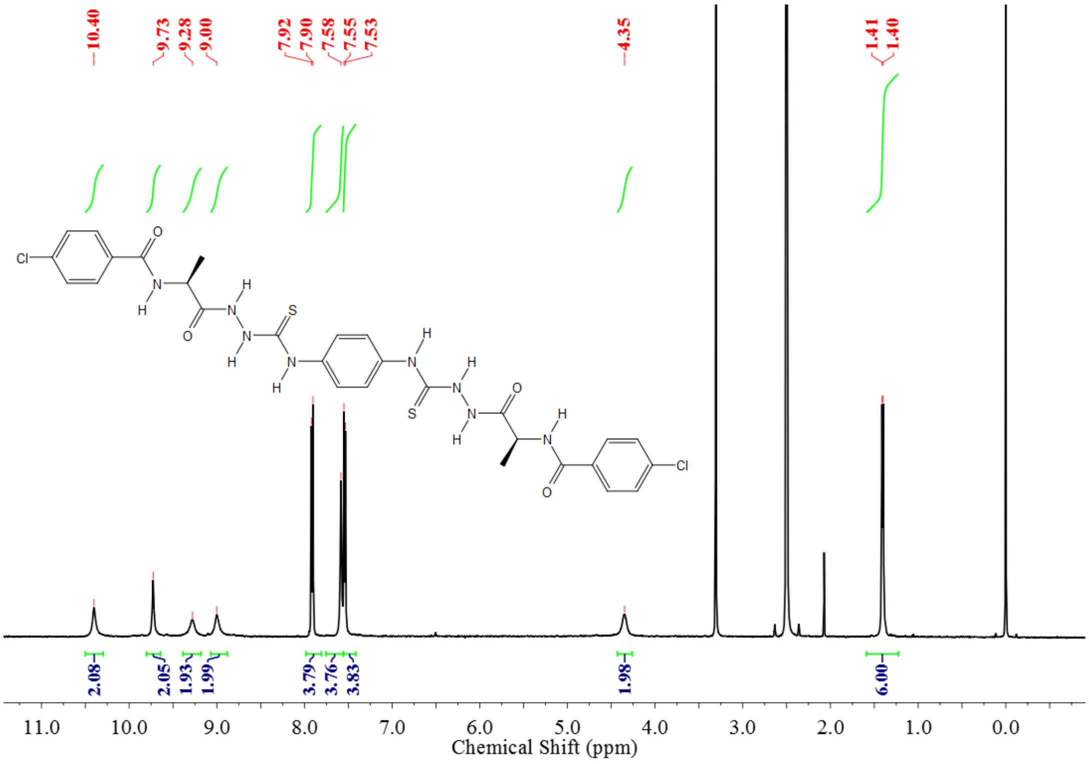

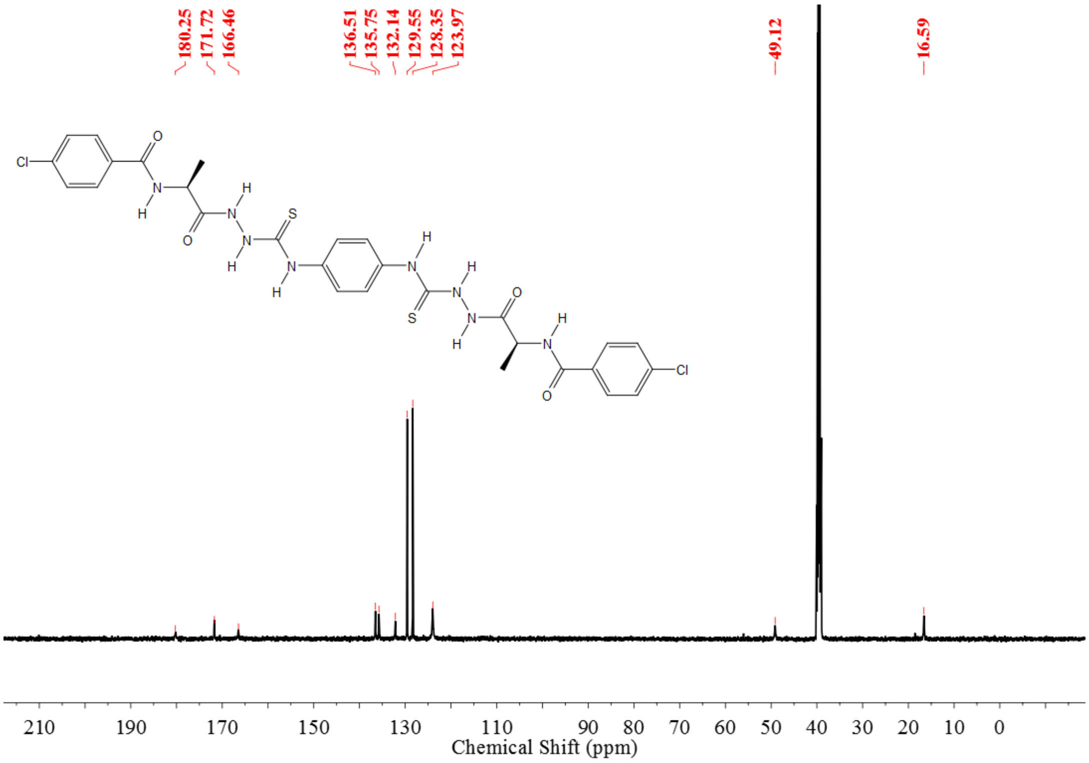

Supplementary Figure 53. ^{1}H and ^{13}C NMR spectra of L,L-**ClA**.

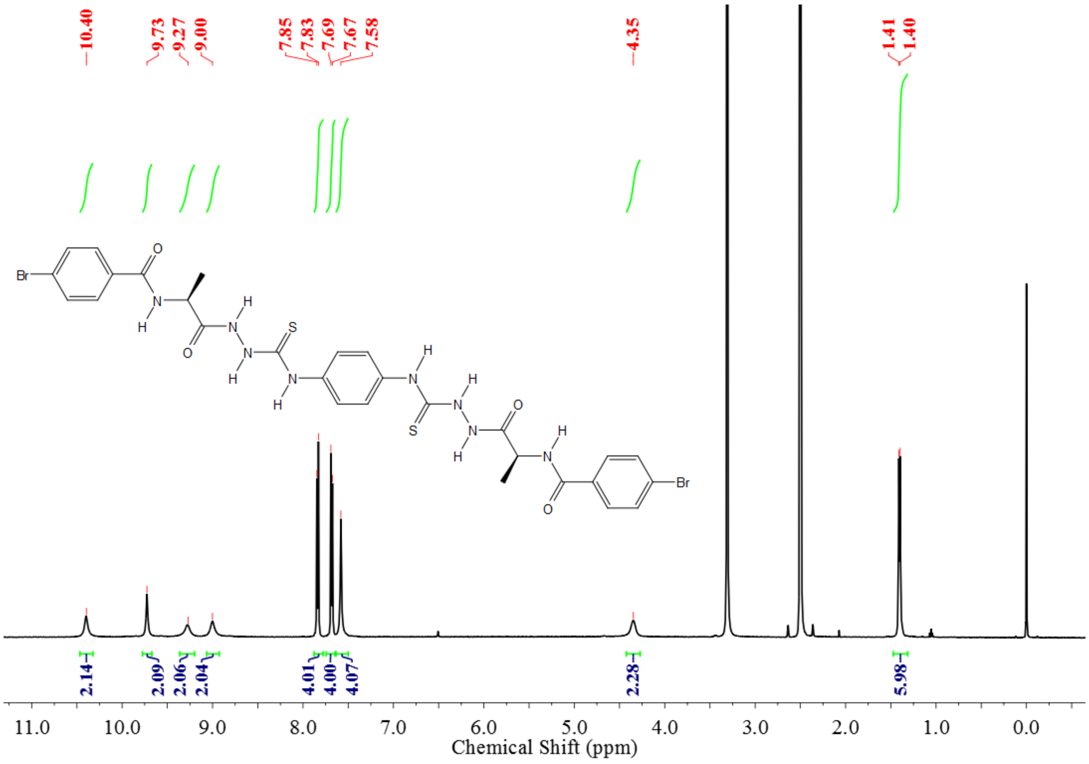

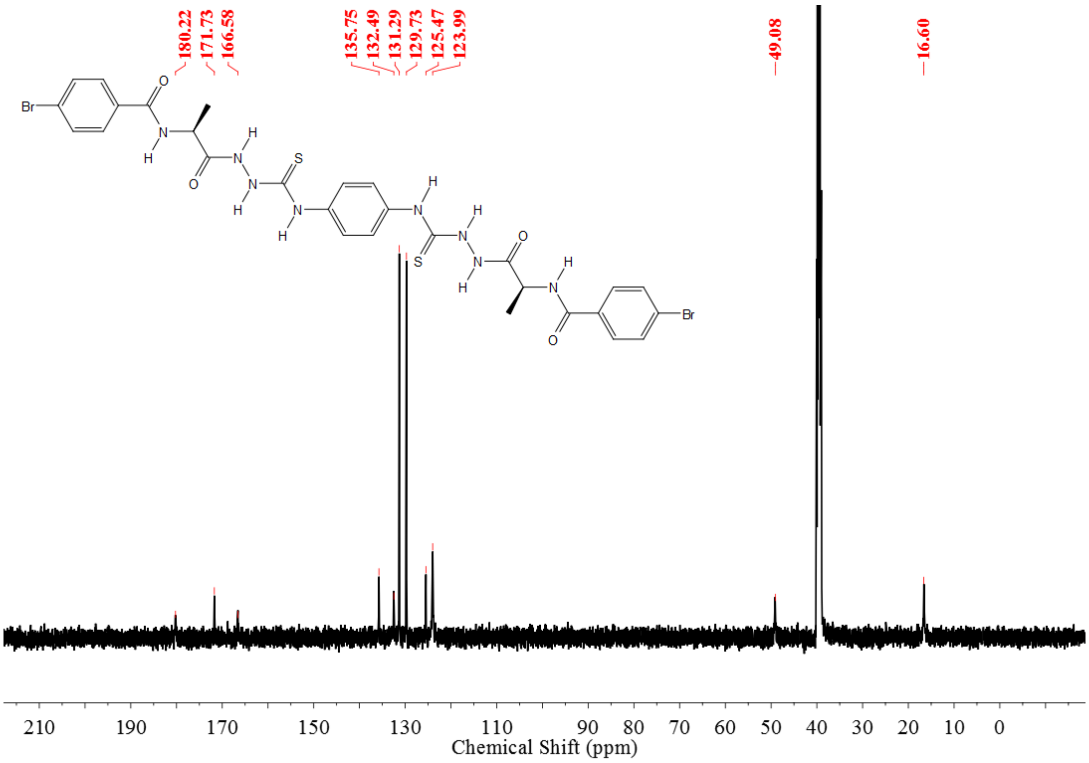

Supplementary Figure 54. ^{1}H and ^{13}C NMR spectra of L,L-**BrA**.

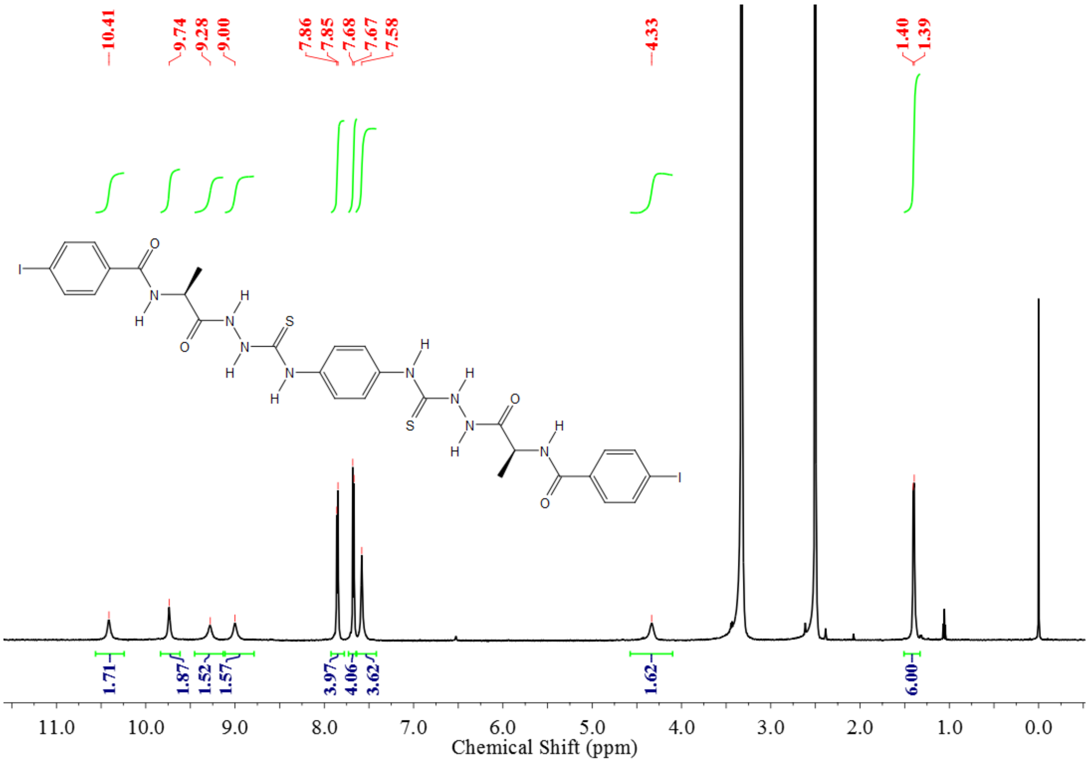

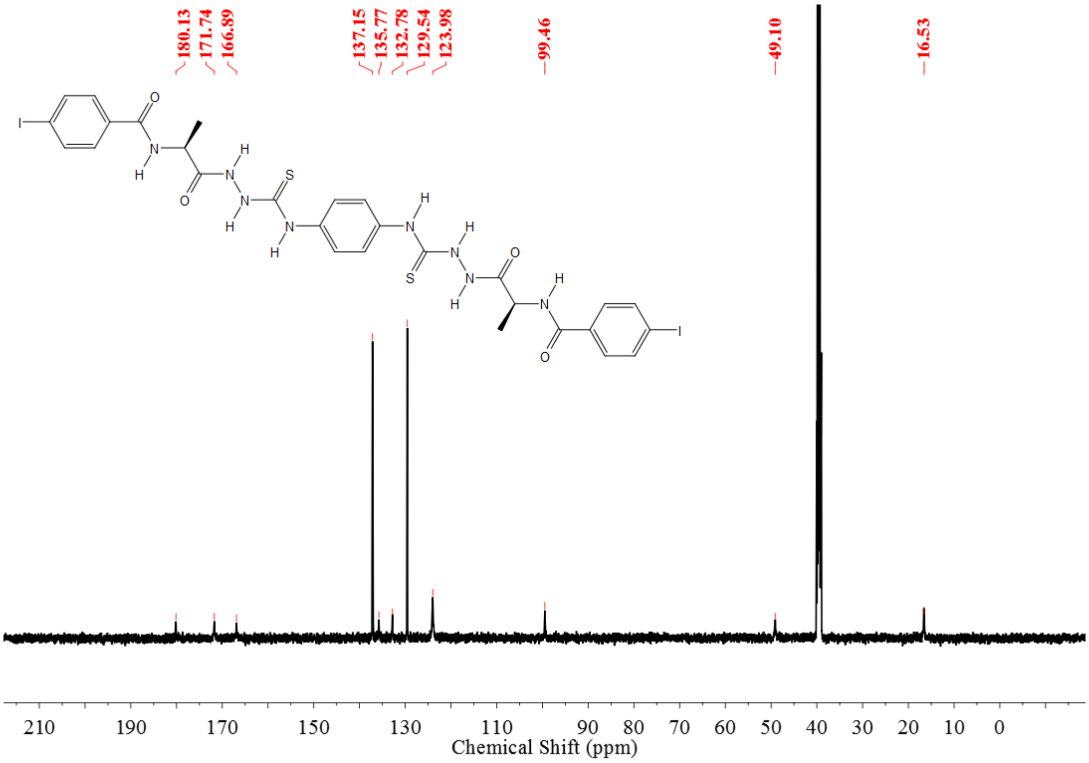

Supplementary Figure 55. ^{1}H and ^{13}C NMR spectra of L,L-**IA**.

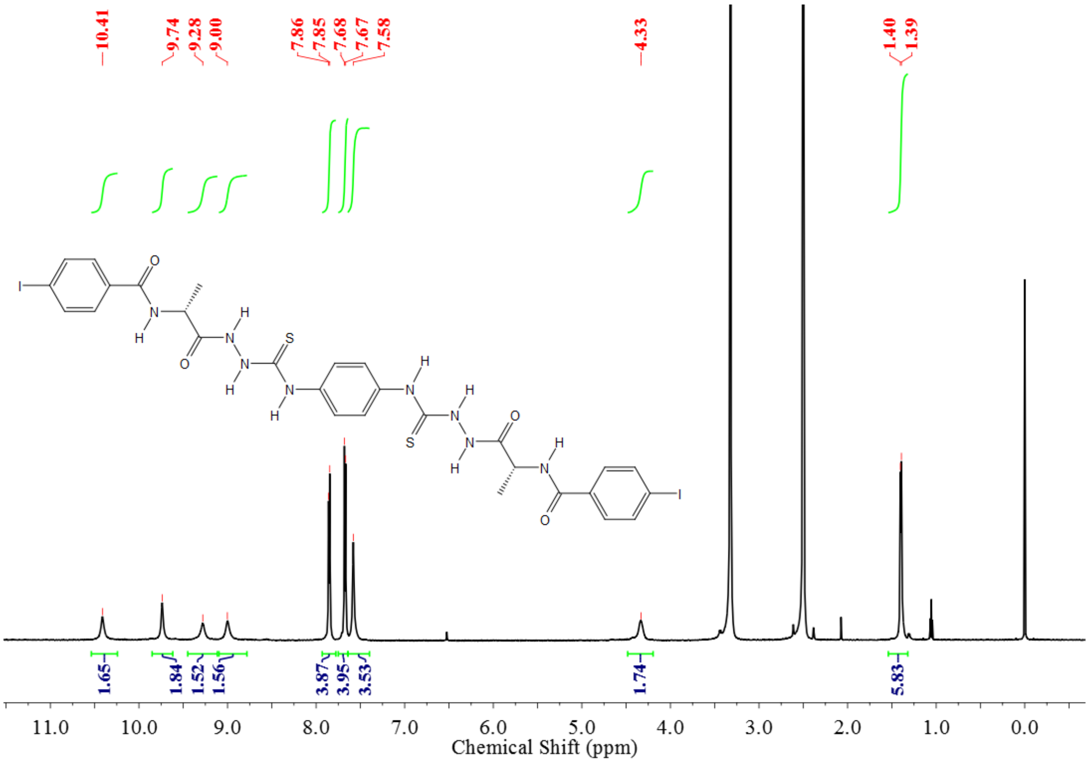

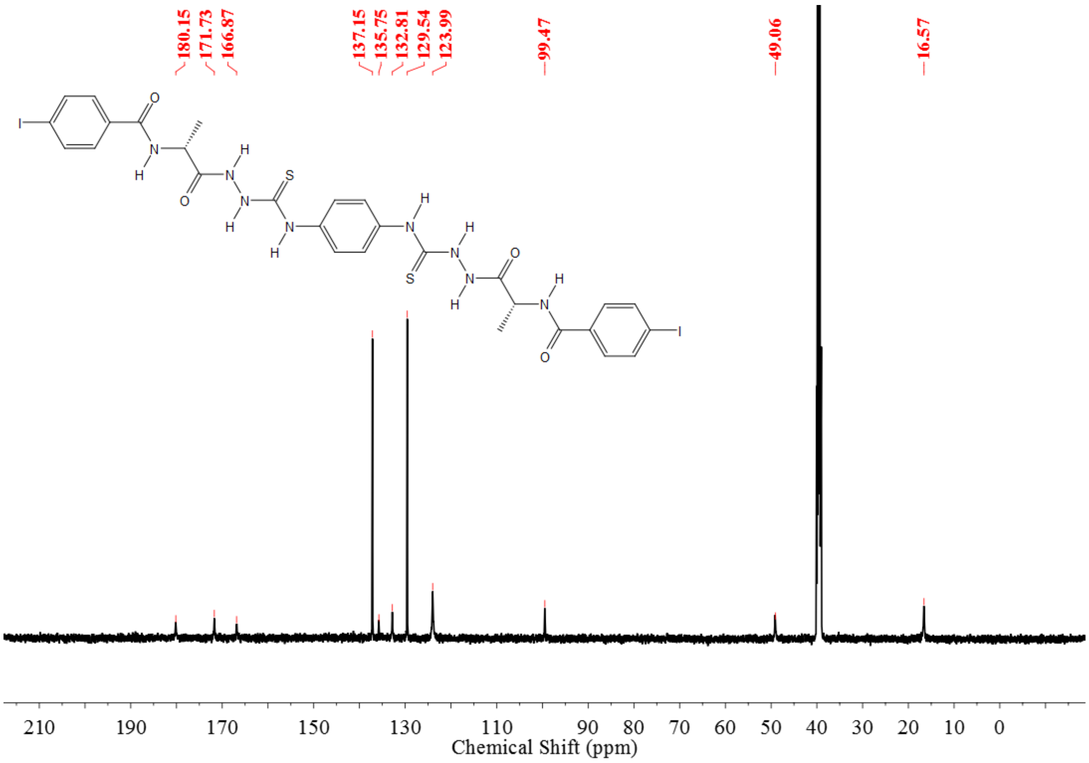

Supplementary Figure 56. ^{1}H and ^{13}C NMR spectra of D,D-**IA**.

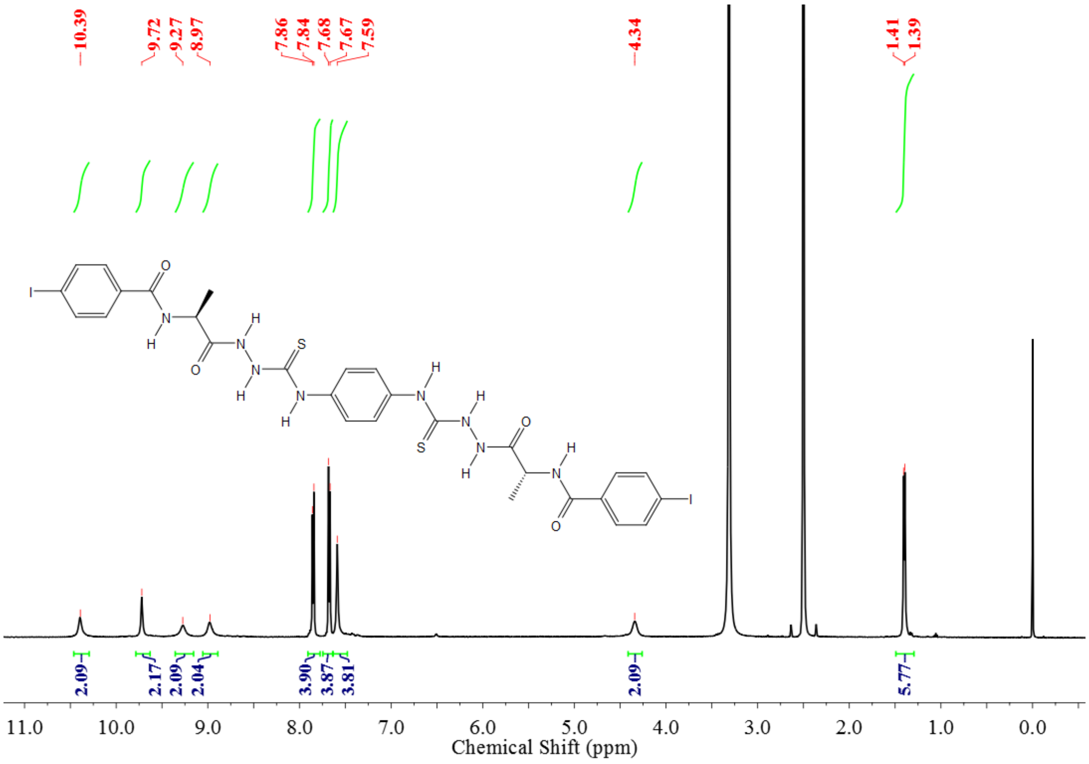

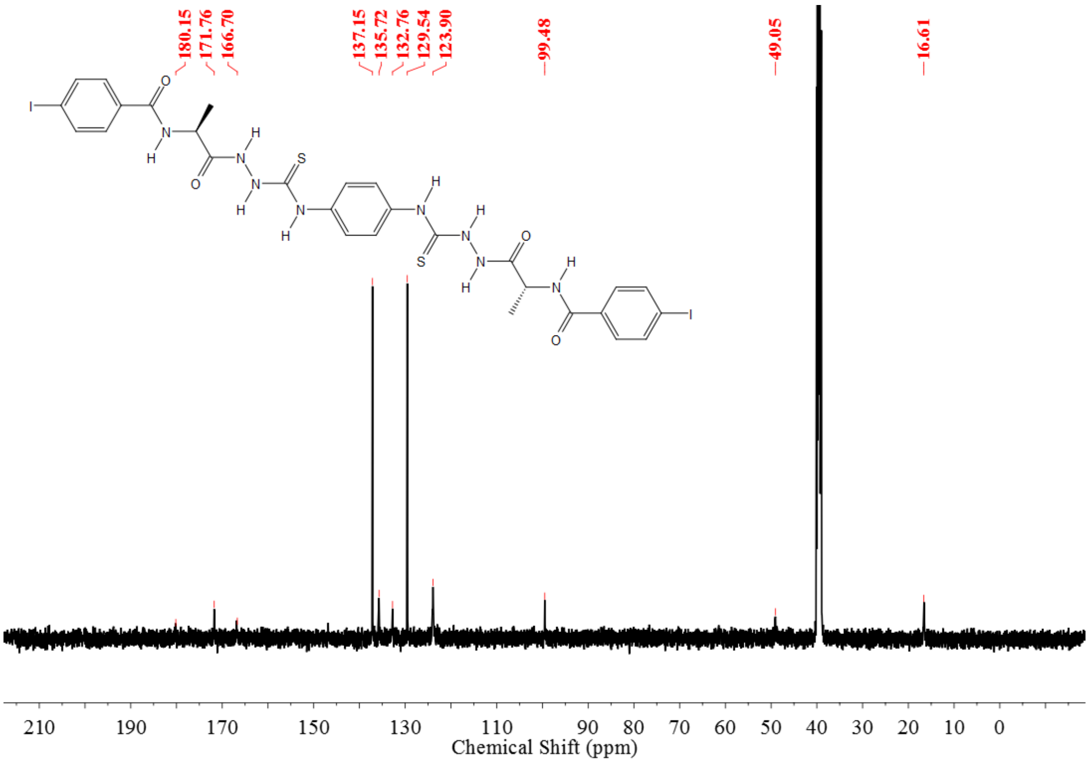

Supplementary Figure 57. ^{1}H and ^{13}C NMR spectra of L,D-**IA**.

Supplementary Tables

Supplementary Table 1. Crystallographic data for L,L-**IA**, D,D-**IA** and L,D-**IA**

Compound reference	L,L-**IA**	D,D-**IA**	L,D-**IA**
Empirical formula	$C_{34}H_{42}I_2N_{10}O_6S_2$	$C_{34}H_{42}I_2N_{10}O_6S_2$	$C_{40}H_{56}I_2N_{12}O_8S_2$
Formula weight	1004.69	1004.69	1150.88
Crystal system	Orthorhombic	Orthorhombic	Triclinic
a, Å	20.5955(4)	20.6103(2)	9.8491(4)
b, Å	8.7308(2)	8.73310(10)	12.1571(5)
c, Å	13.0006(3)	13.00140(10)	12.6102(5)
α, deg	90.00	90.00	86.082(3)
β, deg	90.00	90.00	75.896(3)
γ, deg	90.00	90.00	77.423(3)
V, $Å^3$	2337.71(9)	2340.15(4)	1429.06(10)
Temperature, K	293(2)	293(2)	293(2)
Space group	$P2_12_12$	$P2_12_12$	*P*-1
No. of formula units per unit cell, Z	2	2	1
Radiation type	CuKα	CuKα	CuKα
Absorption coefficient, mm^{-1}	11.805	11.792	9.760
Reflections collected / unique	12985 / 3592 [R(int) = 0.0594]	34969 / 4089 [R(int) = 0.0839]	13059 / 4380 [R(int) = 0.0539]
Final R indices[I>2sigma(I)]	R1 = 0.0448, wR2 = 0.1207	R1 = 0.0444, wR2 = 0.1084	R1 = 0.0740, wR2 = 0.2217
Final R indices (all data)	R1 = 0.0547, wR2 = 0.1375	R1 = 0.0538, wR2 = 0.1132	R1 = 0.0789, wR2 = 0.2321
Goodness of fit on F^2	1.076	1.081	1.038
Flack parameter	-0.015(8)	-0.014(4)	/
CCDC number	1584957	1584955	1584956

Supplementary Table 2. Torsions, types, bond lengths and bond angles of β-turns[4] revealed by the X-ray crystal structures of L,L-**IA**, D,D-**IA** and L,D-**IA**

Compound	L,L-**IA**		D,D-**IA**		L,D-**IA**	
β-turn	**β1**	**β2**	**β1′**	**β2′**	**β3**	**β3′**
φ_{i+1} / °	-64.9(11)	-64.9(11)	62.5(9)	62.5(9)	-60.1(6)	60.1(6)
ψ_{i+1} / °	123.7(8)	123.7(8)	-122.6(7)	-122.6(7)	132.6(5)	-132.6(5)
φ_{i+2} / °	78.1(11)	78.1(11)	-80.1(9)	-80.1(9)	86.0(6)	-86.0(6)
ψ_{i+2} / °	8.0(12)	8.0(12)	-7.1(11)	-7.1(11)	-4.9(6)	4.9(6)
Type	II	II	II′	II′	II	II′
Length / Å	2.223(8)	2.223(8)	2.209(6)	2.209(6)	2.148(3)	2.148(3)
Angle / °	158.9(5)	158.9(5)	159.6(5)	159.6(5)	157.6(3)	157.6(3)

Supplementary Table 3. Geometrical parameters and calculated energies of C−I···S halogen bonding based on crystal structures of L,L-**IA** and D,D-**IA**

Compound	XB	$d_{I\cdots S}$ / Å	θ / °	ΔE / kJ mol^{-1}
L,L-**IA**	**XB1**	3.764(3)	156.3(3)	-23.89
L,L-**IA**	**XB2**	3.764(3)	156.3(3)	-23.89
D,D-**IA**	**XB1′**	3.766(2)	155.6(2)	-23.49
D,D-**IA**	**XB2′**	3.766(2)	155.6(2)	-23.49

Method: DFT wB97XD with the 6-31+G(d, p) basis set for C, H, O, N and S atoms, and LANL2DZ for I atom. The positions of H atoms were optimized whereas those of other heavy atoms are fixed and taken from the crystal structures.

Supplementary Table 4. Comparison between the DNA double helix and the supramolecular double helix in this work

Structural attribute	Double helix of DNA[5]	Double helix in this work
Building blocks	Two complementary polynucleotide strands	*cis*-L,L-**IA** and *cis*-D,D-**IA**
Helical sense	Right-handed: A-DNA, B-DNA<br>Left-handed: Z-DNA	Right-handed: L,L-**IA**<br>Left-handed: D,D-I**A**
One helical pitch	10.4 complementary base pairs (B-DNA)	Two *cis*-L,L-**IA** or *cis*-D,D-**IA**
Pitch length	3.4 nm	2.60 nm
Diameter	2 nm (B-DNA)	1.18 nm
Inter-strand linker	noncovalent hydrogen bonding	covalent *p*-phenylenediamine
Intra-strand linker	covalent phosphate backbone	noncovalent halogen bonding

Supplementary Table 5. Geometrical parameters of C−I···O halogen bonding based on the crystal structure of L,D-**IA**

Compound	XB	$d_{I\cdots O}$ / Å	θ / °
L,D-**IA**	**XB3**	3.184(6)	165.9(2)
L,D-**IA**	**XB3′**	3.184(6)	165.9(2)

Supplementary Table 6. Calculated energies of *trans*- and *cis*-form L,L-**XA** (**X** = **H**, **F**, **Cl**, **Br**, **I**) in gas phase.

Energy (kJ mol^{-1})	*trans*-form	*cis*-form
L,L-**HA**[a]	0.00	8.72
L,L-**FA**[a]	0.00	1.36
L,L-**ClA**[a]	0.00	4.54
L,L-**BrA**[a]	0.00	5.04
L,L-**IA**[b]	0.00	8.37

[a] Calculated energies at B3LYP/6-311G** level. [b] Method: DFT B3LYP with the 6-311G** basis set for C, H, O, N and S atoms, LANL2DZ for I atoms.

Supplementary Methods

Materials. Benzoic acid, 4-fluorobenzoic acid, 4-chlorobenzoic acid, 4-bromobenzoic acid, 4-iodobenzoic acid, 1,4-phenylene diisothiocyanate and acetonitrile for spectroscopy were purchased from Energy Chemical. L- and D-alanine were obtained from GL Biochem (shanghai) Ltd. Acetonitrile-D_3 and dimethyl sulfoxide-D_6, silicon wafers and highly oriented pyrolytic graphite were purchased from Sigma Aldrich. All other starting materials were obtained from Sinopharm Chemical Reagent Ltd.

Synthesis and characterization of L,L- and D,D-XA. To a chilled solution of 4-**X**-substituted (**X** = **F**, **Cl**, **Br**, **I**) benzoic acid or benzoic acid (10.0 mmol) in 20 mL CH_2Cl_2, thionylchloride (10.0 mL) was added. The reaction mixture was then refluxed for 24 h. The solvent was removed by evaporated *in vacuo* to give acyl chloride product **XA-a**. Alanine ethyl ester hydrochloride **AOEt·HCl** (L- or D-, 2.0 g, 13.0 mmol), Et_3N (3.0 mL) and 20 mL $CHCl_3$ were added to a 50 mL round-bottom flask equipped with magnetic stirring. The prepared **XA-a** in 10 mL $CHCl_3$ was added dropwise. The reaction mixture was then stirred at room temperature for 12 h. The solvent was removed by evaporated *in vacuo*. The solid residue was dissolved in EtOAc and then the solution was washed successively with 1% $NH_3.H_2O$, 1% HCl and saturated NaCl solutions. After the solution was dried over anhydrous Na_2SO_4 and concentrated *in vacuo*, solid product **XA-b** (L- or D-) was obtained (80% yield averagely). Excess aqueous hydrazine (85%, 4.0 mL) was added to **XA-b** in EtOH (30 mL) and the mixture was refluxed for 24 hours. The solvent was removed by evaporated *in vacuo*, and the crude product was washed with CH_3CN several times to afford white solid acylhydrazine product **XA-c** (L- or D-) (70% yield averagely). Excess **XA-c** (2.2 mmol) was added to 1,4-phenylene diisothiocyanate **Ph(NCS)$_2$** (1.0 mmol, 0.19g) in CH_3CN (40 mL) and then refluxed for 24 hours. The solvent was removed by filtration, and the crude product was washed with CH_3CN and Et_2O several times to afford pure white solid product **XA** (L,L- or D,D-, 92% yield averagely, Supplementary Figure 1).

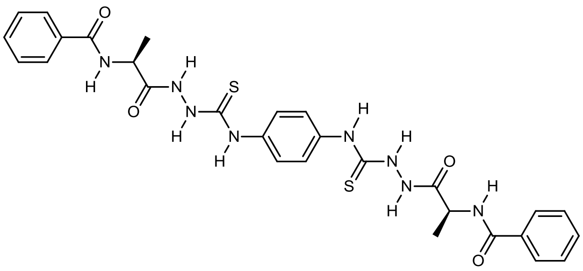

L,L-**HA**

L,L-**HA**. ^{1}H NMR (500 MHz, DMSO-d_6): δ 10.41 (s, 2H), 9.72 (s, 2H), 9.33 (s, 2H), 8.92 (s, 2H), 7.90 (d, J = 7.5 Hz, 4H), 7.62 (s, 4H), 7.54 (t, J = 7.3 Hz, 2H), 7.46 (t, J = 7.5 Hz, 4H), 4.33 (s, 2H), 1.41 (d, J = 6.9 Hz, 6H); ^{13}C NMR (126 MHz, DMSO-d_6): δ 180.14, 171.91, 167.52, 135.75, 133.31, 131.68, 128.25, 127.62, 123.79, 49.13, 16.55; HRMS (m/z): $[M+Na]^+$ calcd. for $C_{28}H_{30}N_8O_4S_2Na$, 629.1729, found, 629.1725.

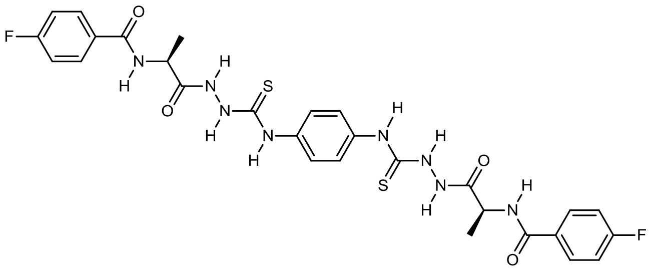

L,L-**FA**

L,L-**FA**. ^{1}H NMR (500 MHz, DMSO-d_6): δ 10.40 (s, 2H), 9.73 (s, 2H), 9.29 (s, 2H), 8.94 (s, 2H), 7.96 (dd, J = 8.2, 5.7 Hz, 4H), 7.59 (s, 4H), 7.29 (t, J = 8.7 Hz, 4H), 4.33 (s, 2H), 1.41 (d, J = 6.8 Hz, 6H); ^{13}C NMR (126 MHz, DMSO-d_6): δ 180.25, 171.81, 166.48, 165.09, 163.11, 135.75, 130.37, 130.30, 139.86, 123.95, 115.28, 115.11, 49.11, 16.59; HRMS (m/z): $[M+Na]^+$ calcd. for $C_{28}H_{28}F_2N_8O_4S_2Na$, 665.1541, found, 665.1531.

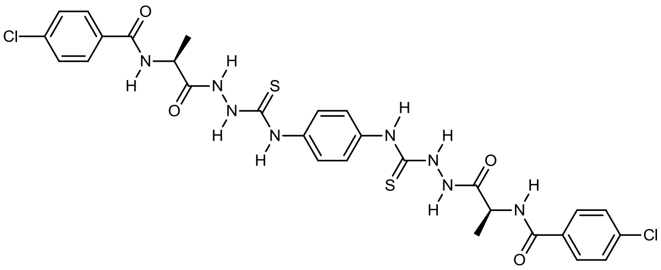

L,L-**ClA**

L,L-**ClA**. ^{1}H NMR (500 MHz, DMSO-d_6): δ 10.40 (s, 2H), 9.73 (s, 2H), 9.28 (s, 2H), 9.00 (s, 2H), 7.91 (d, J = 8.6 Hz, 4H), 7.58 (s, 4H), 7.54 (d, J = 8.5 Hz, 4H), 4.35 (s, 2H), 1.41 (d, J = 7.0 Hz, 6H); ^{13}C NMR (126 MHz, DMSO-d_6): δ 180.25, 171.72, 166.46, 136.51, 135.75, 132.14, 129.55, 128.35, 123.97, 49.12, 16.59; HRMS (m/z): $[M+Na]^+$ calcd. for $C_{28}H_{28}Cl_2N_8O_4S_2Na$, 697.0950, found, 697.0950.

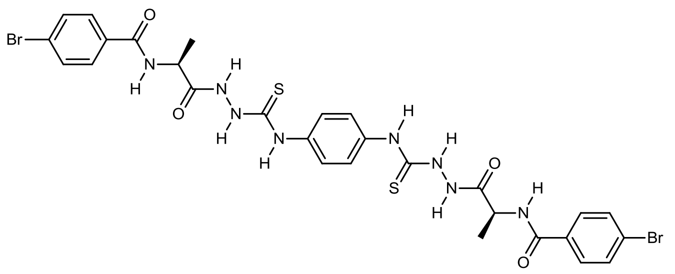

L,L-**BrA**

L,L-**BrA**. ^{1}H NMR (500 MHz, DMSO-d_6): δ 10.40 (s, 2H), 9.73 (s, 2H), 9.27 (s, 2H), 9.00 (s, 2H), 7.84 (d, J = 8.5 Hz, 4H), 7.68 (d, J = 8.5 Hz, 4H), 7.58 (s, 4H), 4.35 (s, 2H), 1.40 (d, J = 7.0 Hz, 6H); ^{13}C NMR (126 MHz, DMSO-d_6): δ 180.22, 171.73, 166.58, 135.75, 132.49, 131.29, 129.73, 125.47, 123.99, 49.08, 16.60; HRMS (m/z): $[M+Na]^+$ calcd. for $C_{28}H_{28}Br_2N_8O_4S_2Na$, 786.9919, found, 786.9928.

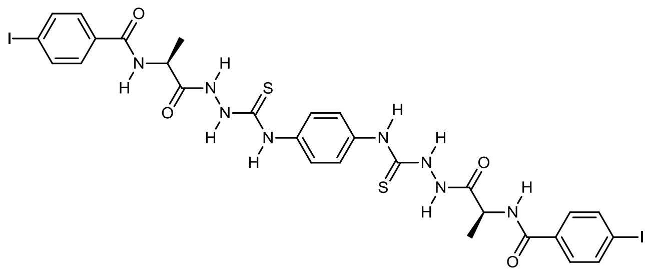

L,L-**IA**

L,L-**IA**. ^{1}H NMR (500 MHz, DMSO-d_6): δ 10.41 (s, 2H), 9.74 (s, 2H), 9.28 (s, 2H), 9.00 (s, 2H), 7.85 (d, J = 8.3 Hz, 4H), 7.67 (d, J = 8.4 Hz, 4H), 7.58 (s, 4H), 4.33 (s, 2H), 1.40 (d, J = 7.0 Hz, 6H); ^{13}C NMR (126 MHz, DMSO-d_6): δ 180.13, 171.74, 166.89, 137.15, 135.77, 132.78, 129.54, 123.98, 99.46, 49.10, 16.53; HRMS (m/z): $[M+Na]^+$ calcd. for $C_{28}H_{28}I_2N_8O_4S_2Na$, 880.9662, found, 880.9662.

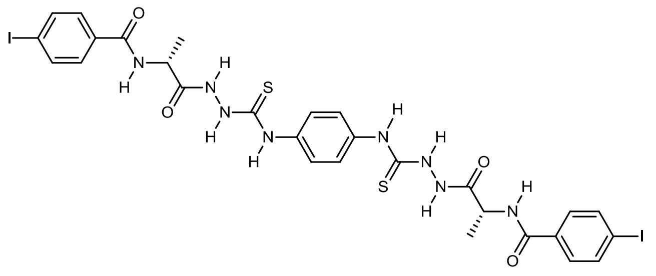

D,D-**IA**

D,D-**IA**. ^{1}H NMR (500 MHz, DMSO-d_6): δ 10.41 (s, 2H), 9.74 (s, 2H), 9.28 (s, 2H), 9.00 (s, 2H), 7.85 (d, J = 8.3 Hz, 4H), 7.67 (d, J = 8.4 Hz, 4H), 7.58 (s, 4H), 4.33 (s, 2H), 1.40 (d, J = 6.9 Hz, 6H); ^{13}C NMR (126 MHz, DMSO-d_6): δ 180.15, 171.73, 166.87, 137.15, 135.75, 132.81, 129.54, 123.99, 99.47, 49.06, 16.57; HRMS (m/z): $[M+Na]^+$ calcd. for $C_{28}H_{28}I_2N_8O_4S_2Na$, 880.9662, found, 880.9671.

Synthesis and characterization of L,D-IA. 1,4-Phenylene diisothiocyanate **Ph(NCS)$_2$** (0.58 g, 3.0 mmol) was dissolved in 20 mL CH_3CN. The suspension of above-mentioned acylhydrazine L-**IA-c** (0.66 g, 2.0 mmol) in 40 mL CH_3CN was added to the **Ph(NCS)$_2$** solution drop by drop, then reflux for 24 h. After filtration, the white solid was purified by silica gel column chromatography (CH_2Cl_2/CH_3OH = 40/1, v/v) to afford desired product L-**IA-d** (0.30 g, yield 29%). Thereafter, L-**IA-d** (0.20 g) and acylhydrazine D-**IA-c** (0.15 g) were added into 20 mL CH_3CN and refluxed for 12 h. After filtration, the white solid was washed with CH_3CN and Et_2O, then dried in vacuum to afford 0.31 g L,D-**IA**, yield 95% (Supplementary Figure 2).

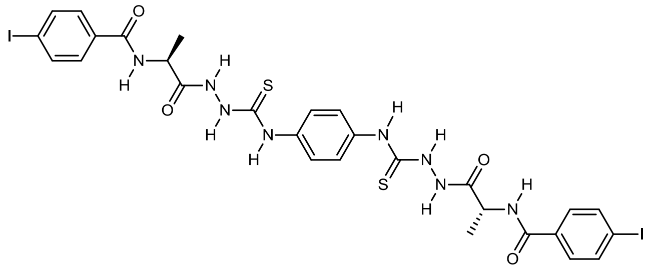

L,D-**IA**

L,D-**IA**. ^{1}H NMR (500 MHz, DMSO-d_6): δ 10.39 (s, 2H), 9.72 (s, 2H), 9.27 (s, 2H), 8.97 (s, 2H), 7.85 (d, J = 8.3 Hz, 4H), 7.67 (d, J = 8.4 Hz, 4H), 7.59 (s, 4H), 4.34 (s, 2H), 1.40 (d, J = 7.0 Hz, 6H); ^{13}C NMR (126 MHz, DMSO-d_6): δ 180.15, 171.76, 166.70, 137.15, 135.72, 132.76, 129.54, 123.90, 99.48, 49.05, 16.61; HRMS (m/z): $[M+Na]^+$ calcd. for $C_{28}H_{28}I_2N_8O_4S_2Na$, 880.9662, found, 880.9671.

Preparation of experimental samples. The single crystals suitable for X-ray diffraction were obtained by slow vapor diffusion of diethylether into DMF solutions of L,L-**IA**, D,D-**IA** and L,D-**IA** (10 mg/mL) at 4 ℃ for one week. The CH_3CN and CD_3CN solutions of all compounds were prepared through sufficient ultrasonic oscillation (60 min) and annealing at 75 ℃ (1 ℃/min) to ensure the dissolution of the solid samples. Absorption measurements were used to confirm the concentrations of all the solutions. For SEM experiments, about 20 μL of the CH_3CN solutions (5 μM) were deposited on silicon wafers. After air-dried for 60 min and platinum coated for 30 s, the samples were analyzed with SEM. For STM experiments, the CH_3CN solutions of L,L-**IA** and D,D-**IA** were diluted into 1 μM. About 10 μL of these solutions were deposited on freshly flatted highly oriented pyrolytic graphite, respectively. The surface was allowed to air-dry for 5 min before analyzing with STM.

Supplementary References

1. Schöpe, H. J., Marnette, O., van Megen, W. & Bryant, G. Preparation and characterization of particles with small differences in polydispersity. *Langmuir* **23**, 11534–11539 (2007).
2. Cao, J. *et al*. C–I···π halogen bonding driven supramolecular helix of bilateral *N*-amidothioureas bearing β-turns. *J. Am. Chem. Soc.* **139**, 6605–6610 (2017).
3. Lee, H. J. *et al.* Role of azaamino acid residue in β-turn formation and stability in designed peptide. *J. Peptide Res.* **56**, 35–46 (2000).
4. Koch, O. Advances in the prediction of turn structures in peptides and proteins. *Mol. Inf.* **31**, 624–630 (2012).
5. Watson, J. D. & Crick, F. H. C. Molecular structure of nucleic acids: a structure for deoxyribose nucleic acid. *Nature* **171**, 737–738 (1953).
